# A new member of the novel, non-core *Brucella* clade: An exotic frog isolate closely related to atypical *Brucella* isolates from recent human brucellosis cases in Australia

**DOI:** 10.1186/s12866-025-04479-2

**Published:** 2025-12-13

**Authors:** Christoph-Martin Ufermann, Dirk Hofreuter, Ashish K. Gadicherla, Cathrin Spröer, Boyke Bunk, Rainer Oehme, Franck Cantet, Stephan Köhler, Sascha Al Dahouk

**Affiliations:** 1 Department of Biological Safety, German Federal Institute for Risk Assessment, Berlin, Germany; 2 Department 1 – Infectious Diseases, Robert Koch Institute, Berlin, Germany; 3 Department of Cell Biology, Neurobiology and Anatomy, Medical College of Wisconsin, Milwaukee, WI USA; 4 Department of Bioinformatics and Databases, Leibniz Institute DSMZ-German Culture Collection for Microorganisms and Cell Cultures, Braunschweig, Germany; 5 State Health Office Baden-Württemberg, Stuttgart, Germany; 6 Institut de Recherche en Infectiologie de Montpellier (IRIM), CNRS, Univ Montpellier, Montpellier, France; 7 Institut de Recherche en Infectiologie de Montpellier (IRIM), CNRS, Univ Montpellier, INSERM, Montpellier, France

**Keywords:** Atypical *Brucella*, Brucellosis, Case report, Frog, Non-core *Brucella*, AMR, NGS, Metabolic phenotyping

## Abstract

**Background:**

Over the past few decades, the *Brucella* genus has seen a significant increase in novel strains that deviate from classical *Brucella* spp. due to their atypical phenotypes. *B. inopinata*, an atypical *Brucella* species first isolated from a patient, was recently found in a White’s tree frog, raising the question of whether amphibians are reservoirs for these emerging human pathogens. Unfortunately, monitoring atypical *Brucella* remains challenging because misidentification with *Ochrobactrum* spp. and *Brucella melitensis* is common when using routine microbiological tests.

**Results:**

In our study, we describe a *Brucella* strain isolated from White’s tree frogs (*Litoria caerulea*) that were initially examined for chytridiomycosis after they had developed dermal abnormalities. Classical microbiological and matrix-assisted laser desorption/ionization time-of-flight mass spectrometry analyses and a species-specific polymerase chain reaction confirmed that isolate CVUAS_1139.3 is an atypical *Brucella* strain. This non-fastidious, fast growing, flagellated, and motile bacterium is not susceptible to lysis by the *Brucella* phages used for typing. Further characterization using the differential metabolic phenotyping approach, revealed that *Brucella* sp. CVUAS_1139.3 could be differentiated from classical *Brucella* spp., as well as from *Ochrobactrum anthropi* and *O. intermedium*, based on its metabolic activity. The substrate utilization patterns may be suitable for a simple and cost-effective diagnostic assay. Phylogenetic analysis positioned *Brucella* sp. CVUAS_1139.3 distant from the classical *Brucella* spp. within the novel, non-core *Brucella* clade. Within this clade, *Brucella* sp. CVUAS_1139.3 shares a close phylogenetic relationship with *B. inopinata* strains and various African bullfrog isolates, and it is most closely related to a recently identified human isolate from Australia. Antimicrobial resistance testing revealed that it is susceptible to antibiotics widely applied in standard treatment regimens. In human THP-1 macrophage-like cells, the replication rate of the novel *Brucella* frog isolate was comparable to that of *B. inopinata*.

**Conclusion:**

In summary, the amphibian-derived strain *Brucella* sp. CVUAS_1139.3 clusters phylogenetically with and is phenotypically alike to previously reported isolates from amphibian hosts and human brucellosis patients within the novel, non-core clade. Our report and other studies suggest that exotic frogs are potential reservoirs for human pathogenic *Brucella* spp., which might pose an underestimated zoonotic hazard for exposed individuals.

**Supplementary Information:**

The online version contains supplementary material available at 10.1186/s12866-025-04479-2.

## Introduction

For over a century, the genus *Brucella* comprised only zoonotic and highly pathogenetic bacteria that cause brucellosis, a disease historically known by various names, such as Malta fever [1], undulant fever [2], and Bang’s disease [3, 4]. These classical *Brucella* species, such as *B. melitensis*, *B. abortus*, and *B. suis*, which have been known for a long time, are characterized by highly similar genomes and are all located in the *Brucella* core clade. Furthermore, their biological features (e.g., growth rate or metabolic activity) define the phenotypes that are typical for *Brucella* [5]. In recent decades, the genus has expanded significantly, with new species and strains emerging that could not be assigned to the previously known classical species. Many of these newly identified strains and species differ from the classical *Brucella* spp. due to their atypical phenotypes, such as rapid growth or motility [6–9] and are therefore referred to as atypical *Brucella* spp. While, for example, the novel *B.* *microti* strains with their atypical phenotypes belong to the core clade, many other atypical isolates reveal more heterogeneous genomes. These cluster phylogenetically outside the core clade, within the non-core clade, such as *B. inopinata* strains [5]. Faster growth than classical *Brucella* species and motility are also common for *Ochrobactrum* spp., such as *O. anthropi* and *O. intermedium*. *Ochrobactrum* spp. are environmental bacteria, some of which have been associated with opportunistic infections in humans [10]. The genus *Ochrobactrum*, the closest phylogenetic relative of *Brucella*, was recently reclassified and merged into the genus *Brucella* [11], a decision that remains highly controversial [12, 13].

Clinical brucellosis cases caused by atypical *Brucella* are rare. Strain BO1, which later became the type strain of the new species *B. inopinata* [14], and BO2 were the first atypical *Brucella* spp. isolated from humans, recovered from patients with a breast implant-associated wound abscess and chronic destructive pneumonia, respectively [15, 16]. Initially misidentified as *Ochrobactrum*, the amphibian-type *Brucella* strain BO3 was the first human isolate with a genome closely resembling that of strain B13-0095 [17], a frog isolate [18]. Reportedly, the patient had contact with amphibians among other animals [17]. An increasing number of phenotypically atypical, yet unclassified *Brucella* sp. isolates from exotic animals have been described, clustering apart from the core clade *Brucella* within the novel, non-core clade. These include strains from Australian rodents [19], African bullfrogs [7, 20], other amphibian hosts such as toads [21], various frog species [22–25], a bluespotted ribbontail ray (strain 141012304) [26], and a panther chameleon (strain 191011898) [27]. Recently, a *B. inopinata* strain was isolated for the first time from a diseased amphibian host, isolate FO700662 from a White’s tree frog [28]. These findings raise further questions about whether amphibians serve as reservoirs for these emerging human pathogens.

Investigating the occurrence of atypical *Brucella* spp. is essential to improve our understanding of their prevalence in animal and human hosts, as well as in the environment. However, monitoring atypical *Brucella* remains challenging, as misidentifications with *Ochrobactrum* spp. and *B. melitensis* occur frequently when using commercially available and automated identification systems, which are partly based on classical microbiological methods (e.g., API and VITEK) and matrix-assisted laser desorption/ionization time-of-flight mass spectrometry (MALDI-TOF MS) [14, 23, 24, 28, 29].

Here, we report a new member of the novel, non-core *Brucella* clade, which was identified during pathological examination of two euthanized exotic frogs (White’s tree frogs). We characterized the isolate using classical microbiological methods for *Brucella* identification and differentiation; applied genus- and species-specific (quantitative) polymerase chain reaction [(q)PCR]; and performed transmission electron microscopy (TEM), MALDI-TOF MS as well as whole-genome sequencing, for in-depth analyses. We analyzed the pathogenic potential of the isolate in an *in vitro *infection assay. Furthermore, we present a differential metabolic phenotyping method that may assist microbiologists in distinguishing between closely related *Ochrobactrum* and both typical and atypical *Brucella*. This, in turn, can enhance disease recognition, surveillance, and control, especially in the current context of taxonomic reclassification and the resulting confusion and debate over how *Brucella* should be defined.

## Materials and methods

### Pathological examination of two exotic frogs as well as primary isolation and identification of *Brucella* sp.

The pathological examination and initial microbiological testing (Additional file 1) of a pair of White’s tree frogs (*Litoria caerulea*, also known as *Ranoidea caerulea*), kept in a zoological collection in Germany, were conducted by the Chemical and Veterinary Investigation Office (Chemisches und Veterinäruntersuchungsamt [CVUA]) in Stuttgart, Germany. A bacterial strain isolated from one of the affected frogs, exhibiting characteristics similar to *Brucella*, was sent to the German Federal Institute for Risk Assessment (Bundestinstitut für Risikobewertung [BfR]) in Berlin, Germany, for in-depth analysis.

### Bacterial cultivation and classical microbiological differentiation

The bacteria were stored at –80 °C in *Brucella* broth (BB) with 40% glycerin. Before the experiments, bacteria were spread on *Brucella* agar (BA) and sub-cultured once at 37 °C with 95% humidity for 24 h, unless stated otherwise.

*Brucella* identification and differentiation were performed as described by Alton et al. [30]. This included growth analysis on BA, *Brucella* selective agar (BSA), sheep blood agar (SBA), and dye-supplemented BA (thionin at 1:25,000, 1:50,000, and 1:100,000 as well as fuchsin at 1:50,000 and 1:100,000), and biochemical tests (urease, oxidase, and catalase), serum agglutination tests (α-A, α-M, and α-R), and phage sensitivity tests with lytic phages (i.e. Iz, BK2, Wb, Tb, R/C, Fi, F25, and F1). Additionally, growth on MacConkey agar (MAC) was analyzed over a 48-h period [31]. Growth in BB was also compared. The initial optical density at 600 nm (OD_600_) was set to 0.05, using fresh colony material in BB. Unless stated otherwise, cultures were grown at 37 °C on an orbital shaker at 150 rpm. An additional 5% CO_2_ was supplied for *B. vulpis* F60 and *B. papionis* F8/08–60. Bacterial growth was monitored over 80 h. The experiments were performed in triplicate. The data are presented as replicate values (symbols) and the mean (line) for each time point, illustrated in an X/Y graph created with GraphPad Prism (v10.1.2).

### Analysis of bacterial motility

Bacterial swarming was analyzed using the motility assay. For this, 2,3,5-triphenyltetrazolium chloride (TTC) soft agar plates were prepared with BB, containing 0.27% (w/v) agar and 50 mg/L TTC for visualization. TTC soft agar plates were inoculated with 10 µL of a bacterial suspension with an OD_600_ of 0.5, prepared from fresh colony material in BB. The plates were incubated at 37 °C and were monitored daily for 96 h.

### Phenotyping based on substrate utilization

Differential metabolic phenotyping, based on substrate utilization, was applied for the microbial differentiation of *Ochrobactrum* and both typical and atypical *Brucella*. We developed this phenotyping approach within the One Health European Joint Project IDEMBRU – Identification of emerging *Brucella* species: new threats for human and animals – which is included in the IDEMBRU toolkit [32, 33]. In brief, bacterial growth was analyzed in a minimal medium, modified from the one described previously [34, 35]. The recipe for this chemically defined minimal medium is provided in Additional file 2.

Single substrates, at a final concentration of 20 mM, were inoculated with bacterial stocks prepared in minimal medium. The initial OD_600_ was adjusted to 0.05. Unless stated otherwise, cultures were grown at 37 °C on an orbital shaker at 150 rpm. *B. abortus* 544, *B. melitensis* 16 M, *B. suis* 1330, *B. canis* RM6/66, *B. ceti* C2/94 and *B. pinnipedialis* B2/94, *B. vulpis* F60, and *B. papionis* F8/08–60 cultures were supplemented with 5% CO_2_. Bacterial growth was monitored for 7 days. The reported values (maximum OD_600_) represent the highest OD_600_ recorded during the monitoring period, from which the initial OD_600_ of the respective culture was subtracted. The experiments were performed at least three times. The data are presented as the mean maximum OD_600_ and illustrated in a grayscale heatmap created with GraphPad Prism (v10.1.2).

### Antibiotic susceptibility testing of *Brucella* sp. CVUAS_11393.3

The diffusion-based epsilometer (E-) test was used for antibiotic susceptibility testing. ETEST® strips (bioMérieux, Marcy-l'Étoile, France) with gradients of the indicated antibiotics (Additional file 3)—including doxycycline, gentamicin, streptomycin, tetracycline, and trimethoprim-sulfamethoxazole—were applied to Mueller–Hinton (MH) agar plates following the guidelines of the Clinical and Laboratory Standards Institute (CLSI) [36]. In brief, fresh colonies were resuspended in saline and adjusted to a McFarland standard of 0.5. The bacterial suspension (100 µL) was spread evenly on MH agar plates and allowed to dry for 10 min. One ETEST® strip per plate was placed aseptically at the center of each plate. The experiments were conducted twice. *Escherichia coli* ATCC 25922 was used in parallel for quality control purposes. The minimum inhibitory concentration (MIC) of each antimicrobial agent against the control and the *Brucella* isolate was recorded (µg/mL) after incubation at 35 °C in ambient air for 20, 24 and 48 h.

### Bacterial identification using MALDI-TOF MS

Sample preparation for MALDI-TOF MS was performed as described previously [37]. Acquisition of mass spectra from duplicate samples and analysis were performed using a MALDI Biotyper™ system (v3.1, Bruker Daltonics, Billerica, MA, USA). The mass spectrum profiles (MSPs) were matched against the ”BDAL” library (11,897 MSPs), the Security-Relevant (SR) database (104 MSPs), and its secondary library ”SR_BBFV”. The Bruker library databases used contain reference spectra classified as *Ochrobactrum* spp. The ”SR”/”SR_BBFV” MSP library allows the identification of all *Brucella* spp. isolates only as *B. melitensis,* without differentiation between *Brucella* species.

### Morphological characterization using transmission electron microscopy (TEM)

Samples for TEM were prepared as follows. *Brucella* sp. CVUAS_1139.3 was cultured on BA and TTC soft agar plates for 24 h. Bacterial cells were collected, washed twice in 25 mM HEPES, and fixed in HEPES with a final glutaraldehyde concentration of 2.5%. Sterility of the bacterial suspension was verified after a 5-min incubation at room temperature by plating an aliquot on BA plates. Samples were stored at 4 °C until further processing for TEM.

For TEM, the sterile bacterial suspension was adsorbed onto 200-mesh carbon coated copper grids for 1 min. After adsorption, excess liquid was removed, and the grid was stained with 2% uranyl acetate for 1 min. Excess stain was removed, and the grids were allowed to dry until imaging.

Imaging was performed using a Jeol 1400 Plus TEM (Jeol GmbH, Freising, Germany) operated at 120 kV. Images were captured using a Veleta G2 camera (Olympus, Hamburg, Germany). A minimum of four distinct areas on the grid was imaged to ensure sample homogeneity.

### Bacterial infection of THP-1 cells

For infection experiments, macrophage-like human THP-1 cells were differentiated with phorbol 12-myristate 13-acetate (PMA) at a final concentration of 100 ng/mL for 2 days. Then, differentiated cells were infected with stationary-phase *Brucella* strains cultivated in Tryptic Soy (TS) broth at a multiplicity of infection of 20 for 45 min. After infection, the cells were washed twice with phosphate-buffered saline (PBS) and incubated in RPMI cell culture medium supplemented with 10% fetal bovine serum and 30 µg/mL gentamicin for at least 1 h. At time points of 2, 4, 24 and 30 h post-infection, host cells were washed once with PBS, lysed with 0.2% Triton X-100, and the number of viable intracellular bacteria was determined by plating serial dilutions onto TS agar. The experiments were performed twice in triplicate. The data were visualized using GraphPad Prism (v10.1.2).

### Genomic DNA extraction

Genomic DNA was isolated from an overnight culture incubated in BB at 37 °C on a horizontal shaker at 150 rpm. The Maxwell® RSC Cultured Cells DNA Kit and the automated Maxwell® RSC 48 DNA purification system (both from Promega, Walldorf, Germany) were used according to the manufacturer’s instructions with slight modifications.

A protocol typically used for Gram-positive bacteria was applied for efficient *Brucella*-DNA extraction using the Maxwell® RSC Cultured Cells DNA Kit. Bacteria were lysed using an enzymatic lysis buffer containing 20 mg/mL lysozyme, 20 mM Tris–HCl, 2 mM ethylenediaminetetraacetic acid (EDTA), and 1.2% Triton. Following a 30-min incubation at 56 °C on a shaker at 300 rpm, RNase A solution (Qiagen, Hilden, Germany; final concentration 2 mg/mL) was added, vortexed, and incubated at room temperature for 5 min. Proteinase K (Qiagen, Hilden, Germany; final concentration 1 mg/mL) and Tris–HCl (pH 8, final concentration 5 mM) were added, vortexed, and incubated at 56 °C on a shaker at 300 rpm for 30 min. The DNA quantity and purity (the OD_260:280_ and OD_260:230_ ratios) were measured using the Qubit dsDNA BR Assay Kit with a Qubit 2.0 fluorometer (both from Invitrogen™, Thermo Fisher Scientific, Darmstadt, Germany) and a NanoDrop spectrophotometer (Thermo Fisher Scientific, Darmstadt, Germany) before use in PCR and sequencing.

### Genus- and species-specific (q)PCR

The genus affiliation of isolate *Brucella* sp. CVUAS_1139.3 was determined by qPCR targeting the *bcsp31* gene and the intergenic element IS711, as described elsewhere [38, 39]. In brief, both *bcsp31* and IS711 qPCRs were performed in a total reaction volume of 25 µL, containing 5 µL of 5 × QuantiFast PCR master mix (Qiagen, Hilden, Germany), 0.3 µM of the respective primer pair, 0.2 µM of the probe, and 2 ng of template DNA. For both qPCRs, the thermal cycling profile included an initial denaturation step at 95 °C for 5 min, followed by 45 cycles of 95 °C for 15 s and 60 °C for 1 min. These assays were performed using a CFX96™ real-time PCR cycler (Bio-Rad, Feldkirchen, Germany).

For molecular species differentiation, the multiplex PCR Bruce-ladder v2.0 was used [40]. The protocol was modified, including a reduced primer concentration [41]. In brief, amplification was carried out in a total reaction volume of 25 µL containing 1 U of Immolase™, 2.5 µL of 10 × ImmoBuffer, and 2 mM MgCl_2_ (all from Meridian Bioscience, Cincinnati, OH, USA), along with 200 µM dNTPs, 0.1 µM of each primer and 1 ng of template DNA. The thermal cycling profile included an initial denaturation step at 95 °C for 7 min, followed by 25 cycles of 95 °C for 35 s and 58 °C for 45 s; final amplification at 72 °C for 6 min. PCR products and the HyperLadder™ 50 bp (Meridian Bioscience, Cincinnati, OH, USA) were separated on a 1.5% agarose gel pre-stained with peqGREEN (1:150,000) in Tris–acetate-EDTA-buffer at 100 V for approximately 2 h.

The primers and probes for the *bcsp31* and IS711 qPCRs [38, 39], as well as the Bruce-ladder v2.0 multiplex PCR [40, 42–44], were synthesized by metabion (Planegg/Steinkirchen, Germany). The sequences are provided in Additional file 4.

### Whole*-*Genome sequencing and in silico analyses

#### Short- and long-read sequencing, quality control and assembly

Sequencing libraries were constructed using the Nextera DNA Flex Library Preparation Kit (Illumina, San Diego, CA, USA). The NextSeq 500/550 Mid Output Kit v2.5 (300 cycles) (Illumina, San Diego, CA, USA) was used for sequencing in paired-end mode with 2 × 149 base pair (bp) reads on an Illumina NextSeq500 instrument. Short-read sequencing data (1,664,912 raw reads) were analyzed using the AQUAMIS (v1.3.8) pipeline [45, 46]. Read quality control and trimming were performed in AQUAMIS with FASTQ (v0.20.1), yielding a total of 1,590,488 trimmed reads containing 245,235,420 bases.

The SMRTbell® template library was prepared according to the manufacturer’s instructions using the SMRTbell® prep kit 3.0 (PacBio, Menlo Park, CA, USA). Briefly, for the preparation of 10-kilobase libraries, 2 µg of genomic DNA was sheared using the Megaruptor® 3 (Diagenode, Denville, NJ, USA) following the manufacturer’s instructions. DNA was end-repaired and ligated to barcoded adapters using components from the SMRTbell® prep kit 3.0. Barcoded DNA samples were pooled equimolarly. The conditions for annealing sequencing primers and binding polymerase to the purified SMRTbell® template were determined with the Calculator in SMRT®link (PacBio, Menlo Park, CA, USA). Libraries were sequenced on the Sequel *IIe* (PacBio, Menlo Park, CA, USA), with one 15-h movie recorded per SMRT cell.

Long-read genome assembly was performed using the “Microbial Genome Analysis” protocol in SMRTlink (v11) with default parameters, except for the target genome size, which was set to 10 megabases. Both chromosomal contigs were assembled as circular and subsequently adjusted to *dnaA* and *parA*, respectively. Error correction was performed by mapping Illumina short reads (see above) onto the finished genome using Burrows-Wheeler Alignment (v0.6.2) in paired-end (sampe) mode with default settings [47], followed by variant and consensus calling using VarScan (v2.3.6) [48]. Genome annotation was based on Prokka (v1.8) [49], followed by manual curation.

The sequencing datasets (BioProject PRJNA1108668) are available at the National Center for Biotechnology Information (NCBI) [50].

#### *In silico* bacterial characterization

In silico bacterial characterization was performed using the BakCharak pipeline (v3.1.2) [51]. Antimicrobial resistance (AMR) genes were identified using BLASTN (v2.16.0 +) [52, 53], as well as the NCBI AMRfinder (v3.12.8) tool in combination with the NCBI AMR gene database (v2024-01–31.1) within BakCharak [54]. Virulence genes were identified in BakCharak using ABRicate (v1.0.1) and the virulence factor database vfdb_brucella_setB (v.2022–08–26) with default parameters, including a minimum gene coverage of 50% [55, 56]. The analyzed strains are listed in Additional file 5.

#### Average nucleotide identity (ANI)-based analysis and phylogeny

Pairwise ANIs were calculated using fastANI (v1.33) with assembled genomes (Additional file 5) [57]. These ANI values are summarized in Additional files 6 and **7**. The data were processed in R (v4.2.0) using the packages matrixcalc (v1.0–6) and ape (v5.6–2) [58–60]. Hierarchical clustering using the unweighted pair group with arithmetic mean (UPGMA) method was performed using the R function hclust. The as.phylo function was used to convert the data into a phylogenetic object, and the resulting Newick file was used to illustrate the phylogenetic tree with the EMBL online tool “interactive Tree Of Life” (iTOL, v6) [61].

#### Single nucleotide polymorphism (SNP)-based analysis and phylogeny

SNPs were identified using the snippySnake pipeline (v1.3.0) [62], which utilizes snippy (v4.6.0) for variant calling [63]. The sequence identifiers of the strains analyzed can be found in Additional file 5. The raw data were trimmed with AQUAMIS, utilizing FASTQ (v0.23.2). The genome sequence of the closest related type strain, *B. inopinata* BO1^T^ (3,366,774 bp), was used as the reference. The core SNPs were used to construct an unrooted phylogenetic tree based on maximum likelihood using IQ-TREE (v2.2.0-beta COVID-edition, 2022–03-03) [64]. Here, branch support values were determined using 1,000 bootstrap replicates with the ultrafast bootstrap (UFboot) method [65] and the Shimodaira–Hasegawa-like approximate likelihood ratio test (SH-aLRT) [66]. The phylogenetic tree was visualized using iTOL (v6) [61].

## Results

### Isolation and identification of a *Brucella* isolate from an exotic frog

A pair of White’s tree frogs (*Litoria caerulea*, also known as *Ranoidea caerulea*) housed in a zoological collection in Germany developed signs of malnutrition, exhibited dermal abnormalities, such as reddened skin with ulcers or subcorneal cysts, and showed additional clinical symptoms consistent with chytridiomycosis (Additional file 1), a devastating fungal disease affecting amphibians [67]. However, microbiological examinations did not confirm chytridiomycosis. Instead, potential pathogens such as *Pseudomonas*, *Acinetobacter*, and bacteria exhibiting colony morphology and growth characteristics similar to *Brucella* were isolated from both frogs (Additional file 1). Suspected *Brucella* colonies were identified from a skin ulcer on Frog 1 and from multiple organs (comprising the liver, kidney, and skin) of Frog 2 (Additional file 1). One isolate from the liver of Frog 2, referred to as isolate CVUAS_1139.3, was selected for further in-depth analysis.

**Fig. 1:**
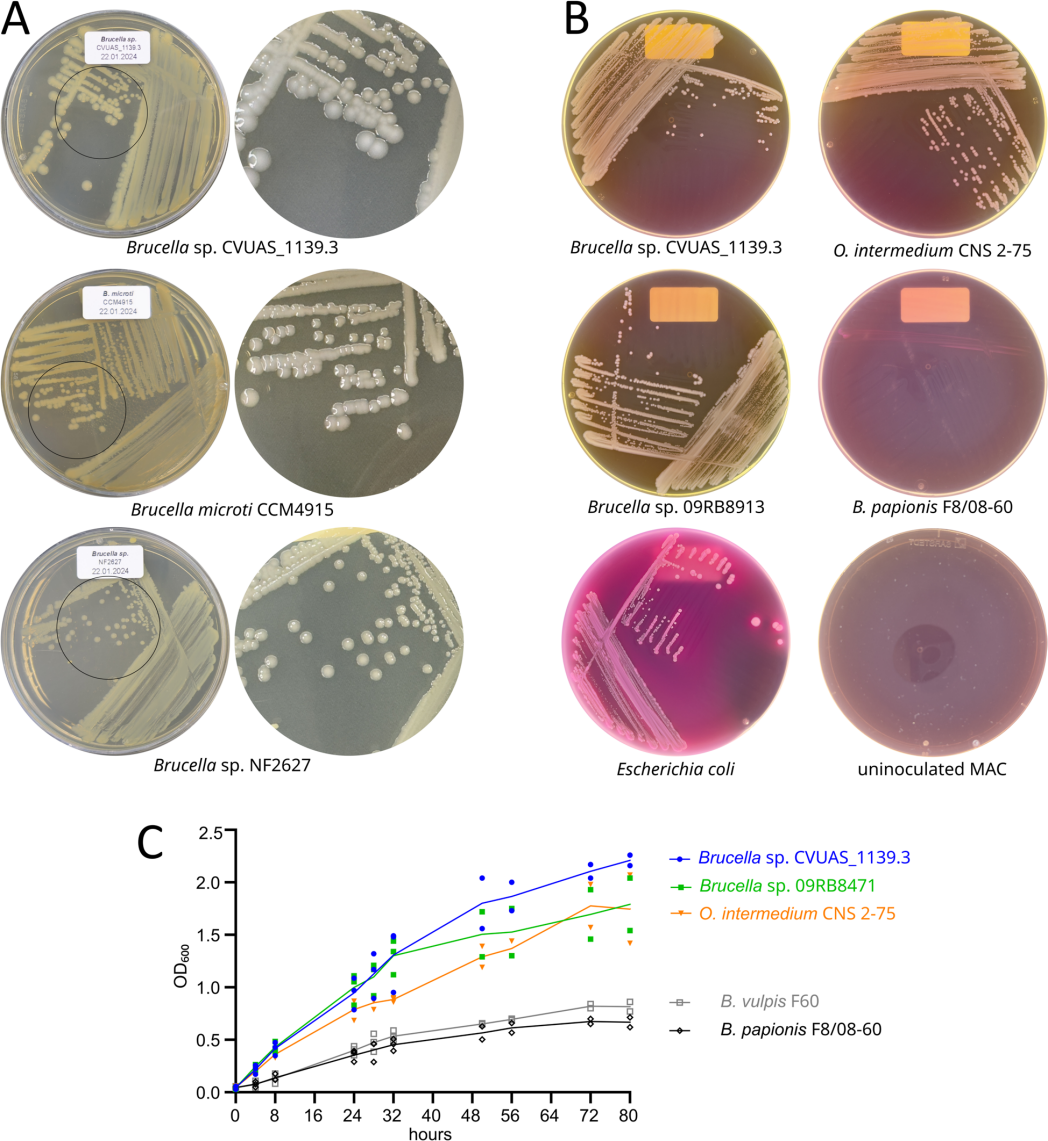
Growth of *Brucella* sp. CVUAS_1139.3 relative to other *Brucella* and *Ochrobactrum* spp. Colony appearance and morphology of *Brucella* sp. CVUAS_1139.3 relative to *B. microti* CCM 4915 (dark beige) and the Australian rodent strain *Brucella* sp. NF2627 (light beige) on *Brucella* agar after incubation for 72 h, shown from the underside (left) and a close-up view (circle inset) from the topside (right; **A**). On MacConkey agar (MAC), *Brucella* sp. CVUAS_1139.3, *O. intermedium* CNS 2–75, and the African bullfrog isolate *Brucella* sp. 09RB8913 display an identical phenotype after incubation for 48 h. *B. papionis* F8/08–60 fails to grow on MAC, while *Escherichia coli* displays lactose-fermenting growth (**B**). *Brucella* sp. CVUAS_1139.3, *Brucella* sp. 09RB8471, and *O. intermedium* CNS 2–75 show comparable rapid growth relative to the *Brucella* species *B. papionis* F8/08–60 and *B. vulpis* F60 in *Brucella* broth. The results are presented as individual values (symbols) per time point from three biological replicates, with the respective mean values indicated by lines (**C**)

We analyzed colony material from isolate CVUAS_1139.3 by using MALDI-TOF MS on a MALDI Biotyper™ equipped with standard MSP libraries (BDAL and SR). The isolate was identified as *B. melitensis*, yielding identification scores ≥ 2.0 for the top three matches (Additional file 8). Classical microbiological characterization of isolate CVUAS_1139.3 revealed a faster growth rate compared with classical *Brucella* spp. when cultured on *Brucella* agar (BA) and *Brucella* selective agar (BSA), both in the presence and absence of supplemented CO_2_. The first colonies on BA were detectable after 18 h, and within 24 h, opaque, circular, and convex colonies, approximately 1 mm in diameter had developed. These colonies maintained a smooth surface after 72 h but exhibited a white-beige coloration, appearing lighter than the beige colonies of *B. microti* CCM4915 and darker than those of *Brucella* sp. NF2627 (Fig. 1A). On MacConkey agar (MAC), isolate CVUAS_1139.3 exhibited growth comparable to the African bullfrog isolate *Brucella* sp. 09RB8913 and *O. intermedium* CNS 2–75, displaying a non-lactose fermenting phenotype accompanied by agar decolorization. In contrast, the fastidious species *B. papionis* did not grow on MAC (Fig. 1B). In *Brucella* broth (BB), isolate CVUAS_11393.3 showed growth comparable to other non-fastidious strains, such as *Brucella* sp. 09RB8471 and *O. intermedium* CNS 2–75, and exceeded the growth rate of the fastidious species *B. vulpis* and *B. papionis* (Fig. 1C). On sheep-blood agar (SBA), the isolate displayed a non-hemolytic phenotype. Similarly to *B. inopinata* BO1 [15] and African bullfrog isolates [7], isolate CVUAS_1139.3 produced H_2_S and exhibited urease, catalase, and oxidase activity. The genus affiliation of *Brucella* sp. CVUAS_1139.3 was ultimately confirmed by *bcsp31* and IS711 qPCRs (data not shown).

### Differentiation of *Brucella* sp. CVUAS_1139.3 – a novel, atypical *Brucella* strain

*Brucella* differentiation via dye sensitivity testing revealed that the growth of *Brucella* sp. CVUAS_1139.3 was unaffected by thionin at 1:25,000 and fuchsin at 1:50,000, the lowest dilutions tested. Colonies of *Brucella* sp. CVUAS_1139.3 were stained with crystal violet, and weak agglutination with monospecific sera, as well as spontaneous agglutination in acriflavine solution was observed. Additionally, *Brucella* sp. CVUAS_1139.3 exhibited resistance to lysis by the bacteriophages commonly used for differentiating classical *Brucella* spp. at all tested concentrations. These characteristics are consistent with other atypical isolates from exotic frogs, such as the African bullfrog strains [7].

**Fig. 2:**
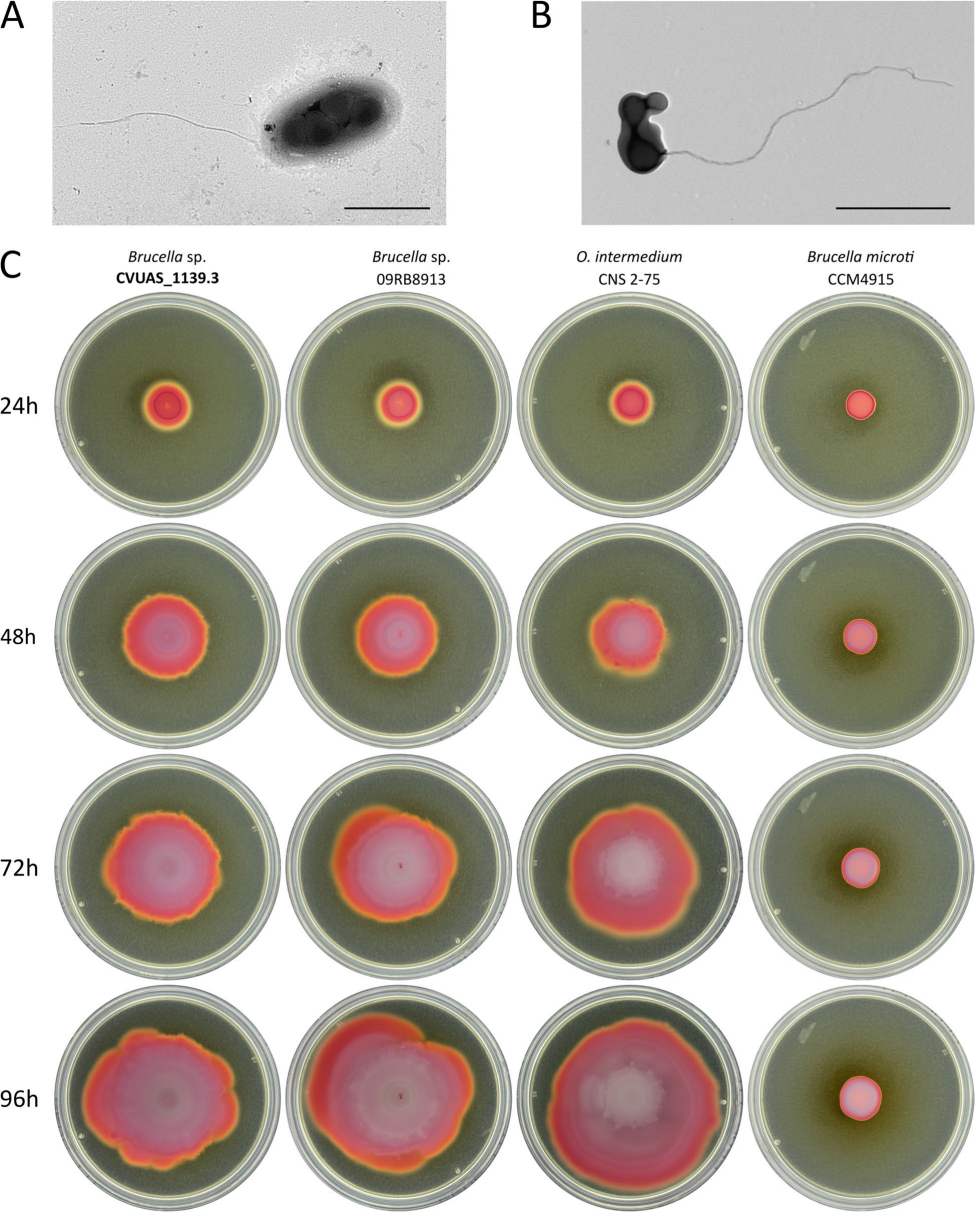
*Brucella* sp. CVUAS_1139.3 is flagellated and motile. Transmission electron microscopy images of individual *Brucella* sp. CVUAS_1139.3 cells with a single monopolar flagellum, sampled from *Brucella* agar (**A**) and 2,3,5-triphenyltetrazolium chloride (TTC) soft agar (**B**), are shown with a 1-µm scale. The motility of *Brucella* sp. CVUAS_1139.3 is compared to that of the motile African bullfrog isolate *Brucella* sp. 09RB8913, *Ochrobactrum intermedium* CNS 2–75, and the non-motile strain *B. microti* CCM 4915 (**C**)

Other atypical *Brucella* spp. have been shown to express a functional flagellum [7]. We performed transmission electron microscopy (TEM) to assess the bacterial cell surface. It revealed that *Brucella* sp. CVUAS_1139.3 is a monopolar flagellated bacterium (Fig. 2A and B). We observed the flagellum in some bacteria isolated from BA (Fig. 2A) and TTC soft agar (Fig. 2B). Since motility is absent in typical *Brucella*, we analyzed the swarming behavior of *Brucella* sp. CVUAS_1139.3. On TTC soft agar plates, *Brucella* sp. CVUAS_1139.3 demonstrated a motile phenotype comparable to that of the African bullfrog strain *Brucella* sp. 09RB8913 or *O*. *intermedium* CNS 2–75 (Fig. 2C).

We used the Bruce-ladder v2.0 assay [40] for molecular species differentiation of *Brucella* sp. CVUAS_1139.3. In this multiplex PCR, *Brucella* sp. CVUAS_1139.3 yielded five amplicon bands (Additional file 9) identical to the band pattern (152, 275, 450, 587, and 774 bp) observed previously in isolates from African bullfrogs, White’s tree frogs, and the bluespotted ribbontail ray [7, 20, 25, 26].

Based on these findings, *Brucella* sp. CVUAS_1139.3 can be classified as atypical; however, it does not match any previously described atypical non-core *Brucella* species.

### Substrate utilization patterns distinguish *Ochrobactrum* and *Brucella* strains

Previously, we demonstrated that metabolic phenotyping using the semi-automated Micronaut system allows for the differentiation of *Brucella* species based on their substrate utilization pattern [68].

**Fig. 3:**
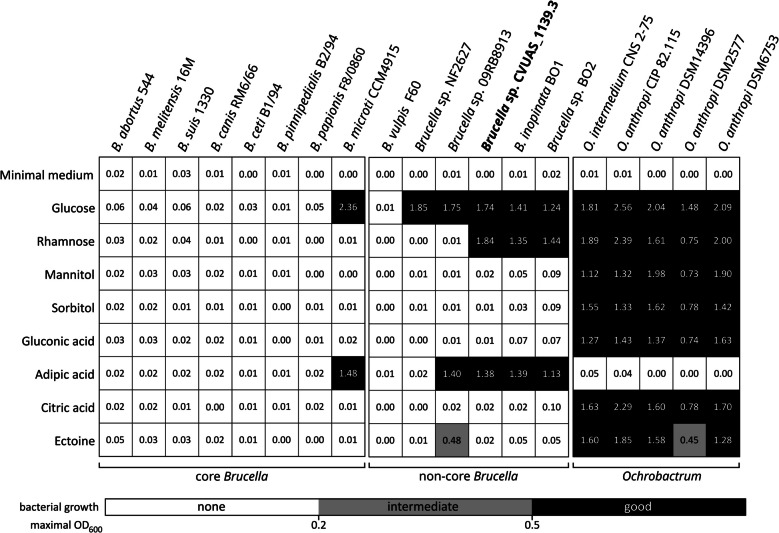
Phenotypic characterization based on the metabolic capacity to grow on selected substrates. Inoculated single substrates (20 mM final concentration) were monitored over a period of 7 days. The data are presented as the mean maximal optical density at 600 nm (OD_600_) of at least three independent experiments. Bacterial growth is categorized as good (black), intermediate (grey), or none (white) based on the following OD_600_ ranges: ≥ 0.5, 0.2–0.49, and ≤ 0.19, respectively

In this study, we employed a simplified growth assay using a defined minimal medium supplemented with potential growth substrates, such as organic acids and sugars, to assess their utilization by *Brucella* and *Ochrobactrum* spp. This approach enabled further phenotypic characterization of *Brucella* sp. CVUAS_1139.3, distinguishing it from typical *Brucella* species as well as from former *O. intermedium* and *O.* *anthropi* isolates based on its substrate utilization pattern (Fig. 3). Notably *Brucella* sp. CVUAS_1139.3 exhibited a substrate utilization pattern identical to that of *B. inopinata* BO1 and *Brucella* sp. BO2: It utilized adipic acid as well as the sugars glucose and rhamnose (Fig. 3).

The fast-growing *Ochrobactrum* spp. were capable of utilizing all tested substrates except adipic acid, demonstrating a non-fastidious substrate utilization pattern (Fig. 3). In contrast, all atypical *Brucella* spp. including *B. microti* CCM 4915—an atypical yet novel core *Brucella* species—were able to grow in the presence of adipic acid. All tested African bullfrog isolates utilized glucose, adipic acid, and ectoine, except for *Brucella* sp. 10RB9213, which also metabolized rhamnose and sorbitol for growth (Additional file 10). None of the typical *Brucella* species, including the majority of the classical type strains (i.e. *B. abortus* 544, *B. melitensis* 16 M, *B. suis* 1330, *B. canis* RM6/66, *B. ceti* B1/94, and *B. pinnipedialis* B2/94) and the novel species *B. vulpis* F60 and *B. papionis* F8/08–60, exhibited growth in minimal medium supplemented with the tested substrates (Fig. 3).

The differential metabolic phenotyping method is effective in distinguishing between *Ochrobactrum* and typical/atypical *Brucella* species. *Brucella* sp. CVUAS_1139.3 was clearly differentiated from *Ochrobactrum* spp. Additionally, *Brucella* sp. CVUAS_1139.3 exhibited a distinct atypical substrate utilization pattern and nutritional requirements, resembling those of some African bullfrog isolates and *B. inopinata* BO1, but markedly differing from classical *Brucella* species.

### *Brucella* sp. CVUAS_1139.3 is a novel, non-core *Brucella* strain

To analyze the phylogenetic positioning of *Brucella* sp. CVUAS_1139.3, we first determined its whole genome sequence using Illumina sequencing. We assessed the quality of the sequencing data by using the in-house developed AQUAMIS pipeline [45, 46]. We also generated PacBio long-read data and combined this information with Illumina short-reads for error correction. The result was a high-quality genome of *Brucella* sp. CVUAS_1139.3, consisting of 3,473,214 bp and a GC content of 57.01%. The genome comprises two chromosomes, measuring 2,202,786 and 1,270,428 bp (BioProject: PRJNA1108668). We annotated a total of 3,343 genes and identified 3,257 coding sequences (CDSs). These include nine ribosomal RNA genes (three of each 5S, 16S, and 23S rRNA) and 58 transfer RNA genes.

**Fig. 4:**
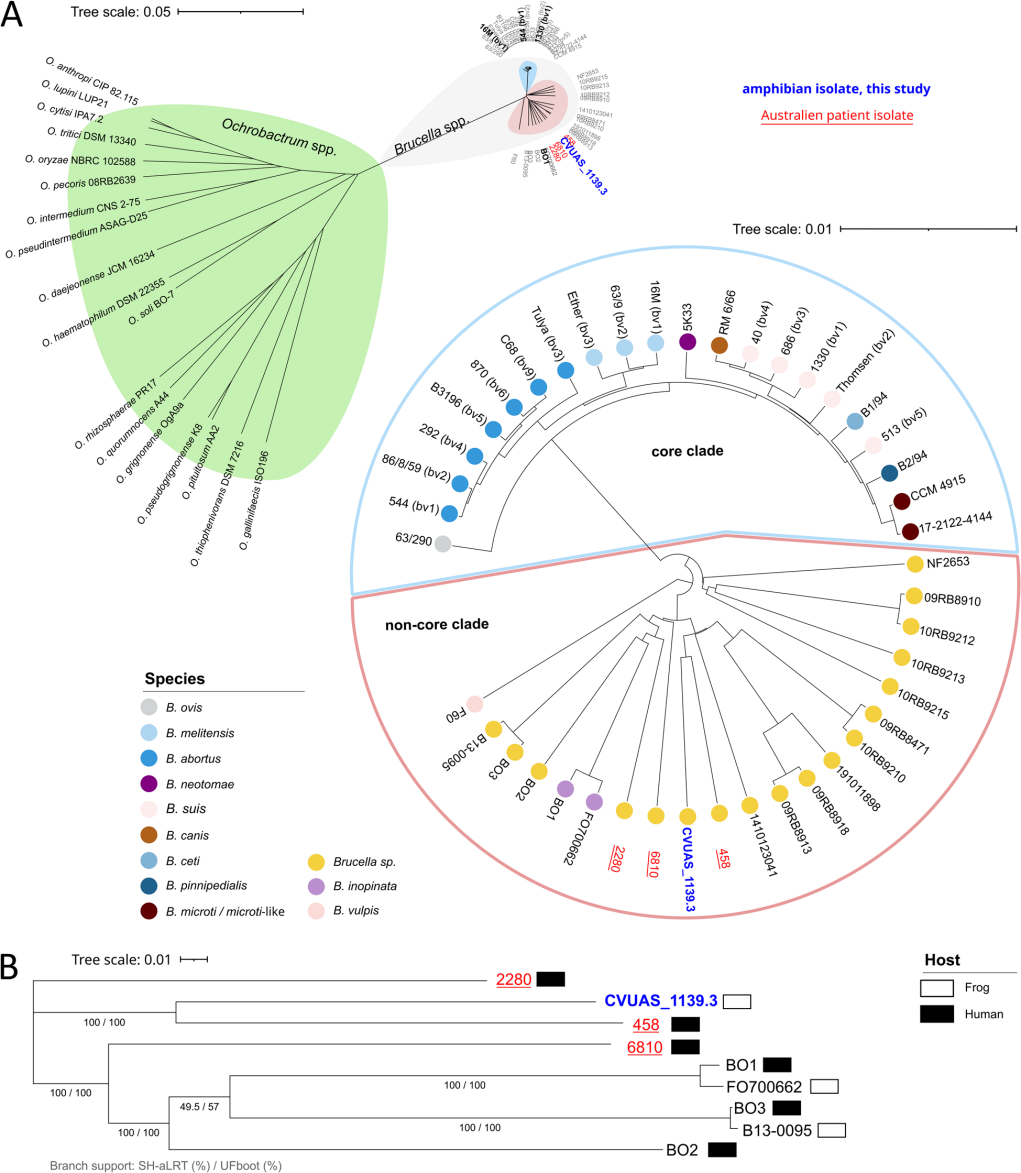
*Brucella* sp. CVUAS_1139.3 is positioned within the non-core *Brucella* clade, in close proximity to human isolates. Pairwise average nucleotide identities, along with hierarchical clustering using the unweighted pair group method with arithmetic mean were applied to position *Brucella* sp. CVUAS_1139.3 (blue, bold) within the *Brucella* clade along the isolates from recent human brucellosis cases in Australia (red, underlined); visualized in both an unrooted and a circular tree (**A**). Maximum likelihood phylogeny based on single nucleotide polymorphisms of a set of non-core *Brucella* (**B**). The tree is unrooted, although the outgroup “2280” is drawn at the root. Strong branch support is confirmed by an Shimodaira–Hasegawa-like approximate likelihood ratio test (SH-aLRT) value ≥ 80% and an ultrafast bootstrap (UFboot) value ≥ 95%. bv, biovar

Genome-to-genome comparison based on average nucleotide identity (ANI) of *Brucella* sp. CVUAS_1139.3 with the *Brucella* type strain *B. melitensis* 16 M revealed 97.43% identity. Comparison with the best-matching type strain, *B. inopinata* BO1, showed 97.90% identity. We observed the highest identity (98.32%) when comparing *Brucella* sp. CVUAS_1139.3 with the human isolate *Brucella* sp. 458 from Australia (Additional file 6). Phylogenetic analysis based on pairwise ANI positioned *Brucella* sp. CVUAS_1139.3 distant from *Ochrobactrum* strains, within the novel, non-core clade basal to the core *Brucella* clade, as shown in the unrooted phylogenetic tree (Fig. 4A). *Brucella* sp. CVUAS_1139.3 is closely related to *Brucella* sp. isolates 458, 6810, and 2280, which were recently isolated from patients in Australia (Fig. 4A and Additional file 11). Next, we identified single nucleotide polymorphisms (SNPs) from a subset of non-core human and frog isolates. For SNP variant calling, we derived a core genome of 2,627,474 bp, corresponding to 78.04% of the reference genome of the type strain *B. inopinata* BO1. Consistent with the ANI analysis, SNP analysis further confirmed that the human isolate *Brucella* sp. 458 is the closest to *Brucella* sp. CVUAS_1139.3 (Fig. 4B).

In summary, we have demonstrated that *Brucella* sp. CVUAS_1139.3, derived from an exotic frog, is indeed a *Brucella* strain located within the novel, non-core clade as determined by ANI. Furthermore, it is closely related to non-core *Brucella* isolates from recent human brucellosis cases in Australia, as confirmed by SNP analysis.

### *Brucella* sp. CVUAS_1139.3 is susceptible to standard antibiotics

It is well established that *Brucella* spp. often contain the multiple peptide resistance factor (*mprF)* [69–71] as well as the multidrug efflux resistance nodulation division (RND) transporter subunit genes *bepC–G* [70, 72, 73]. The MprF protein encodes a phosphatidylglycerol lysyltransferase involved in the lysinylation of phospholipids, thereby contributing to resistance against antimicrobial peptides [74]. The *Brucella* efflux pump C (BepC) protein works in conjunction with the membrane fusion protein RND translocases BepDE and BepFG, which act as substrate transporters, mediating resistance to various substances, including crystal violet and antibiotics, such as ampicillin and norfloxacin [72, 75].

We performed an in silico analysis of antimicrobial resistance (AMR) genes by using BLASTN (v2.16.0 +) [52, 53] and the BakCharak pipeline [51]. It confirmed the presence of the *mprF* gene as well as *bepC*, *bepD*, *bepE*, *bepF*, and *bepG* genes—commonly found in *Brucella*—in the genome of *Brucella* sp. CVUAS_1139.3. We did not identify other potential AMR genes. The *mprF* gene exhibited 100% coverage of the best-matched sequence (AEM20051) with a relative identity of 98.13%. The five *bep* genes showed 100% coverage of the respective sequences (AAN29871.1, AAN29240.1, AAN29241.1, AAN33535.1, and AAN33534.1) with relative identities of 98.46%, 98.99%, 99.05%, 98.78%, and 99.35%, respectively.

Next, we applied a diffusion-based method to assess the in vitro susceptibility of *Brucella* sp. CVUAS_1139.3 to antibiotics. Specifically, we employed ETEST® strips to assess the antimicrobial susceptibility of *Brucella* sp. CVUAS_1139.3, with antibiotics commonly used to treat human brucellosis. These included tetracyclines (tetracycline and doxycycline), aminoglycosides (gentamicin and streptomycin), rifampin, and trimethoprim-sulfamethoxazole [76], as well as ampicillin and norfloxacin, for which susceptibility is influenced by the *bepC–G* gene products, among others. The highest minimum inhibitory concentration (MIC) for each tested antimicrobial agent from two experiments is summarized in Additional file 3 and compared with literature values [15, 23, 24, 77, 78]. Based on the results, *Brucella* sp. CVUAS_1139.3 is susceptible to doxycycline, gentamicin, streptomycin, tetracycline, and trimethoprim-sulfamethoxazole, as the MIC for each antibiotic did not exceed its breakpoint. The MIC for rifampin was comparable to isolates reported in other studies. Although the MIC for norfloxacin has not been previously analyzed in novel *Brucella*, it is within a similar range of MICs reported for classical *Brucella* species [79, 80]. The MIC for ampicillin is consistent with that observed for other novel strains, suggesting typical transporter (BepC/BepDE/BepFG) function without significant susceptibility or resistance of *Brucella* sp. CVUAS_1139.3 (Additional file 3).

Taken together, in silico and in vitro AMR analysis of *Brucella* sp. CVUAS_1139.3 revealed that this isolate contains typical AMR genes found in other *Brucella* spp. Additionally, *Brucella* sp. CVUAS_1139.3 was shown to be susceptible to antibiotics commonly used to treat human brucellosis.

### *Brucella* sp. CVUAS_1139.3 harbors virulence genes known from other *Brucella* strains and displays high proliferative activity in human macrophages

**Fig. 5:**
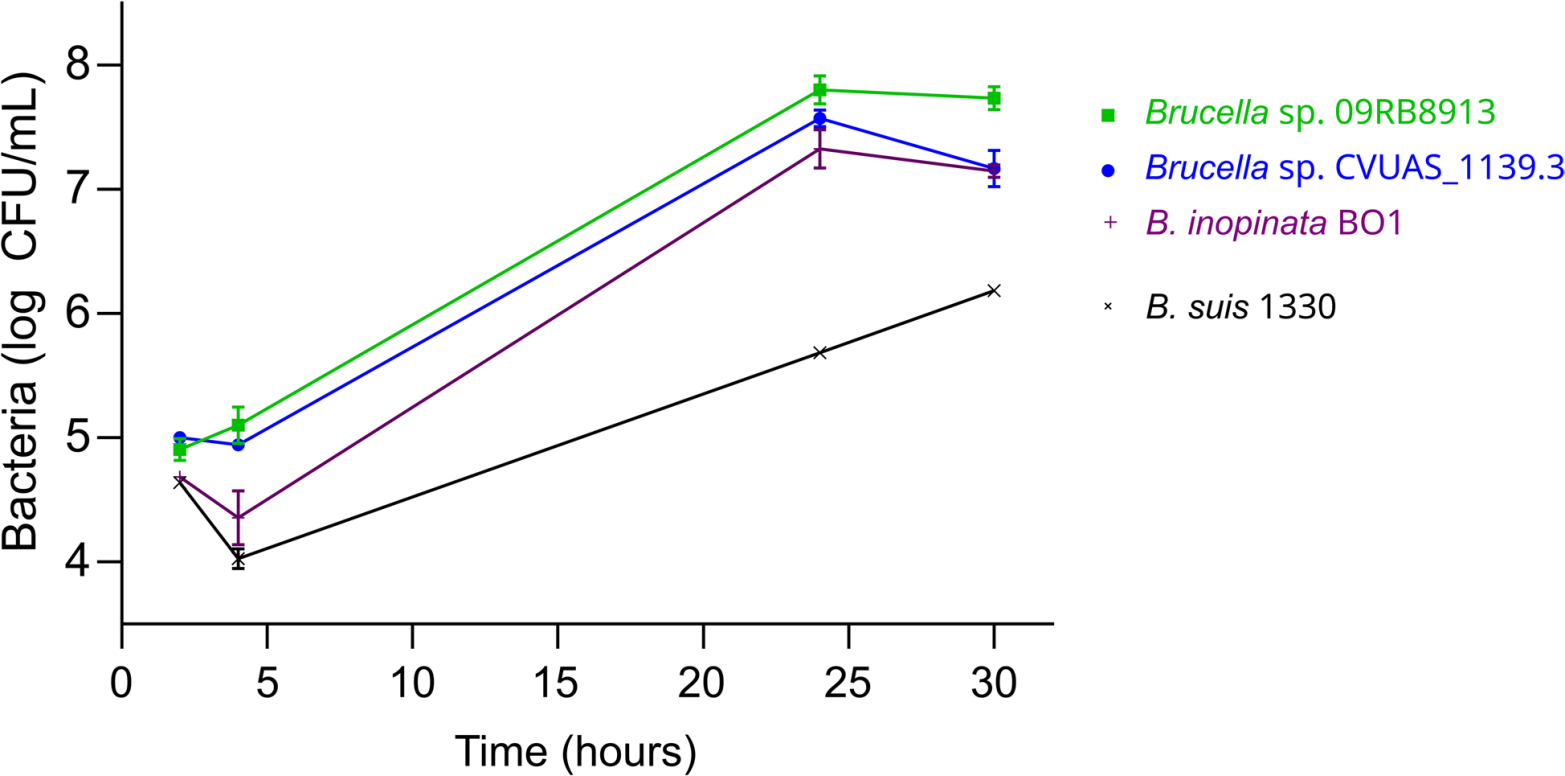
Intracellular replication of novel and classical *Brucella* spp. in human THP-1 macrophage-like cells. Bacterial replication of *Brucella* sp. CVUAS_1139.3 compared with novel *Brucella* spp., the African bullfrog strain *Brucella* sp. 09RB8913, *B. inopinata* BO1 and the classical *Brucella* representative *B.* *suis* 1330. The experiment was performed twice, each time in technical triplicate, yielding comparable results (see Additional file 14). The results from one experiment are presented as the mean ± standard deviation. Error bars that are smaller than the symbol are not shown. CFU, colony-forming unit

In silico analysis of the genome of *Brucella* sp. CVUAS_1139.3 identified a set of 58 genes that encode virulence factors previously described in other *Brucella* strains, including the complete *virB* operon (Additional files 12 and 13). The type 4 secretion system (T4SS) encoded by the *virB* operon is essential for *Brucella* survival within macrophages [81–83]. To evaluate the functionality of the T4SS in *Brucella* sp. CVUAS_1139.3, infection experiments were conducted using PMA-differentiated human THP-1 macrophage-like cells. *Brucella* sp. CVUAS_1139.3 was compared to *Brucella* strains BO1, 09RB8913 and 1330 (Fig. 5 and Additional file 14). The intracellular replication capacity of *Brucella* sp. CVUAS_1139.3 was similar to that of the novel strains *Brucella* sp. 09RB8913 and *B. inopinata* BO1. In contrast, all three novel strains replicated more rapidly and reached higher intracellular bacterial loads than the classical reference strain *B. suis* 1330.

Phage typing revealed that *Brucella* sp. CVUAS_1139.3 is not sensitive to lysis by commonly used lytic bacteriophages (i.e., Iz, BK2, Wb, Tb, R/C, Fi, F25, and F1). These phage typing results suggest that the lipopolysaccharide (LPS) of *Brucella* sp. CVUAS_1139.3 differs from that of typical *Brucella* strains. We performed a bioinformatic analysis, in which we considered genes to be absent if their sequence coverage was below 50%. Accordingly, *Brucella* sp. CVUAS_1139.3 lacks several (n = 13) immunomodulatory genes involved in LPS biosynthesis (i.e., *gmd, manA*_*O-Ag*_*, manC*_*O-Ag*_*, per, pmm, wbdA, wbkA, wbkB, wbkC, wboA, wbpZ, wzm*, and *wzt*), while other LPS-associated genes (e.g., *manB*_*core*_ and *wbpL*) are present. With respect to the LPS gene content, the human isolate *Brucella* sp. 458 from Australia is identical to *Brucella* sp. CVUAS_1139.3. The other two recently identified human *Brucella* isolates from Australia (*Brucella* sp. strains 2280 and 6810) are also missing most of these LPS genes, with the exception of *manA*_*O-Ag*_*, manC*_*O-Ag*_, and *wbpZ* (Additional files 12 and 13). In contrast, the genomes of *B. inopinata* strains BO1 and FO700662 contain the complete set of these LPS biosynthesis genes [28].

First described in *B. suis*, the *bmaC* gene encodes an autotransporter located in the outer membrane that is involved in adhesion to host cells, but is not essential for macrophage infection [84]. Similar to the genome of *Brucella* sp. CVUAS_1139.3, several non-core *Brucella* spp. genomes analyzed in this study (i.e., BO1, BO2, 458, 2280, and FO700662) lack the *bmaC* gene, whereas in others (including strain 09RB8913) *bmaC* gene coverage exceeds 99% (Additional files 12 and 13).

The *btpA* gene encodes the *Brucella* Toll/interleukin-1 receptor (TIR)-domain-containing protein BtpA, which has been reported to interact with host cells [85]. In the genome of *Brucella* sp. CVUAS_1139.3, the *btpA* gene shows 100% sequence coverage and identity to the reference (WP_002965528.1), also known as *btp1* in *B. abortus* [85]. The *btpA* gene is absent in the genomes of the other novel *Brucella* strains analyzed in this study (Additional files 12 and 13).

In summary, both in silico and in vitro analyses of *Brucella* sp. CVUAS_1139.3 demonstrated that this newly described isolate is comparable to other novel and atypical *Brucella* strains, such as the African bullfrog strains, with respect to the presence of virulence factors and its pathogenic potential in in vitro infection models.

## Discussion

In this study, we reported and characterized *Brucella* sp. CVUAS_1139.3, which was isolated from a pair of diseased White’s tree frogs. Exotic frogs are increasingly recognized as common hosts for atypical *Brucella* spp., as frogs and other amphibians, such as toads, have repeatedly been shown to harbor atypical *Brucella* isolates over the past decade [6, 7, 18, 20, 22–25, 28, 86, 87]. It remains unclear whether *Brucella* spp. act exclusively as pathogens in amphibians or whether they colonize these hosts asymptomatically, acting as opportunistic pathogens. Amphibians are particularly vulnerable to infections by opportunistic pathogens and commensal microbiota when exposed to environmental stressors, such as fluctuations in temperature and humidity [88]. We cannot confirm whether the frogs described herein were exposed to such stressors, as the origin of the frogs was not disclosed; it remains uncertain whether they were wild-caught, imported and quarantined, part of an established collection, or recently transferred to a new enclosure.

The amphibian skin microbiome consists of a diverse array of bacteria, including members of the phyla Proteobacteria, Bacteroidetes, and Actinobacteria [89, 90], some of which contribute to the sex-specific scent of frogs [91] and may aid in protection against pathogens. The Actinobacteria genus *Streptomyces*, [92] and the Proteobacteria genera *Pseudomonas* and *Acinetobacter* have been reported as components of the skin microbiome in frogs [93] and have been shown to offer protection against chytridiomycosis caused by *Batrachochytrium dendrobatidis* [94–97]. Nevertheless, *Pseudomonas* can cause bacterial dermatosepticemia (red-leg syndrome) in frogs [88, 98], and *Acinetobacter* has been implicated in mass mortality events in salamanders [99]. In other case reports, *Brucella* has been identified as the causative agent of specific pathologies, including abscesses and skin lesions [21, 28, 86, 100]. The skin ulcer of Frog 1 was colonized by *Brucella*; however, Frog 2 did not present with such a specific pathology. Thus, it is unclear whether the poor health observed in both White’s tree frogs was solely due to colonization by *Brucella* spp., as opportunistic pathogens (e.g., *Pseudomonas* spp. and *Staphylococcus* sp.) were also identified. Nevertheless, *Brucella* likely contributed to the poor health status of Frog 2, as it was isolated from various organs of this individual, showing strong growth in skin and liver samples, as well as weak growth in the kidney. The frogs were of opposite sexes and similar in age, so it is likely that they were housed together as a pair in the same enclosure. However, we cannot confirm whether both frogs were colonized by the same *Brucella* strain, as only one specimen (from the liver of Frog 2) was available for in-depth analysis.

Improving our understanding of the prevalence of atypical *Brucella* in hosts and the environment is crucial for assessing the risk of exposure to these pathogens. However, monitoring atypical *Brucella* is challenging. For example, Australian cane toads with spinal arthropathy were investigated in a report by Shilton et al. [101]. Spinal arthropathy, also known as spondylitis, is a symptom commonly caused by *Brucella* spp. [102, 103]. In the diseased toads, bacteria were isolated and identified as *O. anthropi* using the API 20 NE test system [101]. Their affiliation with the genus *Brucella* was excluded due to their motile phenotype and growth on MAC [101]. Motility and growth on MAC are important criteria for excluding typical *Brucella* spp. during the microbiological identification process, but they fail to exclude atypical *Brucella* spp. [7]. Consistently, the isolate described in our study, *Brucella* sp. CVUAS_1139.3, could not be differentiated from *Ochrobactrum* based on growth on MAC and TTC soft agar. The alleged *O. anthropi* isolates from cane toads reported by Shilton et al. [101] were reanalyzed at a time when the first reports of atypical *Brucella* from amphibians had emerged. In that 2016 study, these isolates were ultimately identified as atypical *Brucella* based on *recA* gene analysis [6]. Initial misidentification of atypical *Brucella* as *Ochrobactrum* spp. using commercial test systems is not un-common [14, 16, 20, 23–26, 28, 29]. Moreover, classical *Brucella* (e.g., *B. melitensis* and *B. suis*) are occasionally misidentified as *Ochrobactrum* [104–108]. In summary, differentiation between *Brucella* and *Ochrobactrum* is not always flawless. The metabolic phenotyping approach we developed involved a set of five substrates, enabling the discrimination between *Ochrobactrum* and both atypical and typical *Brucella* strains. Growth of the typical *Brucella* isolates tested in our study, most of which are classified as risk group 3, was not supported by any of the substrates in the minimal medium. The atypical *Brucella* isolates grew in the presence of either glucose or adipic acid. Adipic acid, along with sugar derivatives (mannitol and sorbitol), gluconic acid, and citric acid were the key substrates for discriminating between atypical *Brucella* and *Ochrobactrum* (e.g., *O. intermedium* and *O. anthropi*), given that *Ochrobactrum* spp. grew with all of the substrates except adipic acid. Their capacity to utilize a wide range of substrates, resulting in distinct metabolic profiles, may reflect the nutritionally diverse niches in which these bacteria naturally occur, with *Ochrobactrum* spp. being environmental organisms and atypical *Brucella* spp. being pathogens associated with various hosts.

Accurate identification of pathogenic organisms in diagnostic settings is essential for downstream testing (e.g., antimicrobial susceptibility testing), the implementation of appropriate biosafety measures (handling *Ochrobactrum* can occur at lower biosafety levels, whereas handling *Brucella* typically requires specialists at reference laboratories/centers), and ultimately for correct antibiotic treatment, making it critical from a public health perspective. Recently, the genus *Ochrobactrum* has been merged into the genus *Brucella* [11]. This inclusion has sparked a science-backed debate within the *Brucella* community, supported by clinical and veterinary microbiologists who urge professionals to continue applying the “*Brucella*/*Ochrobactrum*” nomenclature [12, 13, 70, 109, 110]. Adopting the new “only *Brucella*” nomenclature increases the risk of confusing specimens from and infections by pathogenic *Brucella* spp., which cause brucellosis, with those caused by opportunistic *Ochrobactrum* spp., thus complicating the application of accurate biosafety precautions and treatment regimens.

Brucellosis is typically treated with a combination of tetracyclines (tetracycline and doxycycline), aminoglycosides (streptomycin and gentamicin), and rifampin; depending on the clinical context, trimethoprim-sulfamethoxazole may also be included [76]. Treatment regimens for opportunistic *Ochrobactrum* infections are not standardized and are largely influenced by the resistance profiles of the respective isolates [10]. Resistance of *Ochrobactrum* spp. to gentamicin and trimethoprim-sulfamethoxazole—antibiotics commonly used in the treatment of brucellosis—has been documented in cases of endophthalmitis and bacteremia caused by multidrug-resistant *O. intermedium* [111] and *O. anthropi* [112].

Various methods, including broth microdilution and diffusion-based assays (e.g., disc-diffusion and the E-test), are employed for antimicrobial susceptibility testing of *Brucella* isolates [15, 24, 73, 113, 114]. Currently, no quality control MIC ranges for E-tests are available; therefore, we used the MICs obtained via broth microdilution, as outlined in the CLSI guidelines [36, 115], as the reference standards. According to the CLSI interpretive criteria, *Brucella* sp. CVUAS_1139.3 is susceptible to doxycycline, gentamicin, streptomycin, tetracycline, and trimethoprim-sulfamethoxazole. Moreover, the MICs and susceptibility profile of *Brucella* sp. CVUAS_1139.3 closely resemble the antimicrobial susceptibility testing results (Additional file 3) obtained from other atypical amphibian *Brucella* sp. isolates (n = 27) and *B. inopinata* BO1 [15, 23]. These findings indicate that, despite their phylogenetic proximity to *Ochrobactrum*, atypical non-core *Brucella* do not harbor clinically relevant multidrug-resistance mechanisms. Since both typical and atypical *Brucella* are zoonotic pathogens, the emergence of antibiotic resistance would significantly hamper successful treatment of human brucellosis.

In general, clinical cases of brucellosis caused by atypical *Brucella* isolates (such as BO1, BO2, and BO3) are rarely reported in the literature [15–17]. However, the zoonotic potential of atypical *Brucella* spp. was recently highlighted by Scholz et al. [28], who described isolate FO700662, the first *B. inopinata* strain isolated from an exotic frog. Both *B. inopinata* FO700662 and the isolate described in this study, *Brucella* sp. CVUAS_1139.3, were obtained from diseased White’s tree frogs. With respect to their basic microbiological characteristics, *Brucella* sp. CVUAS_1139.3 and *B. inopinata* FO700662 appear indistinguishable from one another, a phenomenon observed in many atypical *Brucella* spp. [5]. Both exhibit rapid growth even in the absence of supplemental CO_2_; are positive for catalase, oxidase, and urease; produce H_2_S; and are non-hemolytic [28]. In addition, *B. inopinata* FO700662 was tested using the API 20NE system, revealing the ability to metabolize adipic acid but not citric acid—two characteristics that are informative for distinguishing *Brucella* from *Ochrobactrum* based on substrate utilization patterns, as demonstrated in the present study.

Based on MALDI-TOF MS, we identified *Brucella* sp. CVUAS_1139.3 as *B. melitensis* with a score of 2.2, whereas *B. inopinata* FO700662 was identified as *Ochrobactrum* sp. with an identical score. However, whole-genome analysis revealed clear genetic differences between the two isolates. While *B. inopinata* FO700662 showed high similarity to *B. inopinata* BO1, our exotic frog–derived isolate, *Brucella* sp. CVUAS_11393, could not be assigned to any currently described species, with *Brucella* sp. 458 identified as its closest relative. The latter is one of three recently reported human isolates—*Brucella* sp. 2280, 6810, and 458—obtained from patients in coastal regions of Queensland, Australia, and isolated from lymph node, blood, and lung tissue (Additional file 11). Apart from the associated metadata and the availability of high-quality genome sequences, no further information is currently available regarding the clinical cases or the microbiological properties of these isolates.

Although there is currently no direct evidence of an epidemiological link, our phylogenetic analysis connects an amphibian-derived isolate (*Brucella* sp. CVUAS_11393) with a human-derived isolate (*Brucella* sp. 458), reinforcing the notion that amphibians may serve as reservoirs for atypical *Brucella* spp*.*

## Supplementary Information

Additional file 1. Case summary of two White’s tree frogs (*Litoria caerulea*^§^) examined at the CVUAS.

Additional file 2. Composition of minimal medium and substrates used for differential metabolic phenotyping.

Additional file 3. Minimum inhibitory concentrations (MICs) determined for *Brucella* sp. CVUAS_1139.3 using the E-test, compared with literature values.

Additional file 4. List of primers and probes used in this study.

Additional file 5. Sequence data used in *in silico* analyses.

Additional file 6. Average nucleotide identities (ANIs) between *Brucella* sp. CVUAS_1139.3 and all analyzed *Brucella* spp. and *Ochrobactrum* spp. strains.

Additional file 7. Pairwise average nucleotide identities of all analyzed strains.

Additional file 8. MALDI Biotyper™ identification reports for *Brucella* sp. CVUAS_1139.3.

Additional file 9. Bruce‑ladder v2.0 results for *Brucella* sp. CVUAS_1139.3.

Additional file 10. Differential metabolic phenotyping of exotic frog-derived *Brucella* sp. isolates.

Additional file 11. Metadata for recently identified Australian *Brucella* sp. isolates that cluster within the novel, non-core clade.

Additional file 12. Virulence factor genes and their coverages identified in a selection of novel *Brucella* strains.

Additional file 13. A detailed overview of all virulence factor genes and antimicrobial resistance genes identified in a selection of novel *Brucella* spp.

Additional file 14. Intracellular replication of novel and classical *Brucella *spp. in human THP-1 macrophage-like cells.

## Data Availability

The datasets generated in this study are available in the repositories of the National Center for Biotechnology Information (NCBI) under the following accession numbers PRJNA1108668 (BioProject), SRR28969979 (Sequencing Read Archive) and CP159274-CP159275 (GenBank).
