# Supplementary material for: A new member of the novel, non-core Brucella clade: An exotic frog isolate closely related to atypical Brucella isolates from recent human brucellosis cases in Australia
Source: BMC Microbiol. 2025 Dec 13;25:790. doi: 10.1186/s12866-025-04479-2 (PMC12701591; doi:10.1186/s12866-025-04479-2)
Supplement: Supplementary file 1 — Additional file 1. Case summary of two White’s tree frogs (Litoria caerulea§) examined at the CVUAS. [file 12866_2025_4479_MOESM1_ESM.pdf]

Additional file 1 Case summary of two White's tree frogs (*Litoria caerulea*[§]) examined at the CVUAS.

		Frog 1	Frog 2
**A**	Sex | weight | age	♀ | 91 g | unknown, possibly very old	♂ | 66 g | unknown, possibly very old
**B**	abdominal skin	Reddened	Reddened
	Skin ulcer	Several (≤ 2 mm) on the left arm, left and right side of the abdomen; one deep and large (6 mm) ulcer on the left flank	None
	Edema	None	Subcutaneous and abdomen
	Nutritional status	Malnutrition	Malnutrition
**C**	Body cavity	Inconspicuous	Filled with a clear fluid, severe effusion (ascites)
	Kidneys and urinary bladder	Inconspicuous	Renal calculi in the ureter, strongly filled bladder with reddish/brownish fluid
	Skin	No additional findings	Subcorneal cysts filled with erythrocytes and serum
	Other organs	Inconspicuous	Inconspicuous
**D**	Skin	*Staphylococcus* sp. + ;<br>*Stenotrophomonas maltophilia*, +	*Brucella* sp. [1] ++
	Skin ulcerations	*Brucella* sp. [1] ++ ;<br>cestode larvae in larger ulcer	N/A
	Skin cysts	N/A	Without signs of pathogens
	Lung	-	-
	Liver	Unspecified mixed bacterial culture +	***Brucella* sp. [1#] ++** ;<br>unspecified mixed bacterial culture +
	Kidney	*Acinetobacter* sp. + ;<br>*Pseudomonas aeruginosa*;<br>unspecified bacteria ++	*Brucella* sp. [1] + ;<br>additional mixed bacterial cultures +
	Intestinal tract	-	*Streptomyces* sp. [2] ++ ;<br>yeasts ++ ;<br>additional mixed bacterial cultures ++
	Bladder	N/A	*Acinetobacter* sp. + ;<br>*Escherichia coli* + ;<br>*Streptomyces* sp. [2] +;<br>*P. aeruginosa* +++ ;<br>unspecified bacteria +++

A – metadata; **B** – clinical signs; **C** – pathology; **D** – microbiological findings by culture* and microscopic** methods; CVUAS, Chemisches und Veterinäruntersuchungsamt Stuttgart, Germany.

*Specific *Salmonella* spp. enrichment and **direct virus particle detection by electron microscopy was negative for pooled organ samples from both frogs; [1]microaerophilic; [2]anaerobic cultivation; bacterial growth scale: - none, + weak, ++ strong, +++ massive; N/A – not applicable; [§]also known as *Ranoidea caerulea;* [#]origin of isolate **CVUAS_1139.3**
