# Supplementary material for: A new member of the novel, non-core Brucella clade: An exotic frog isolate closely related to atypical Brucella isolates from recent human brucellosis cases in Australia
Source: BMC Microbiol. 2025 Dec 13;25:790. doi: 10.1186/s12866-025-04479-2 (PMC12701591; doi:10.1186/s12866-025-04479-2)
Supplement: Supplementary file 2 — Additional file 2. Composition of minimal medium and substrates used for differential metabolic phenotyping. [file 12866_2025_4479_MOESM2_ESM.pdf]

Additional file 2 Composition of minimal medium and substrates used for differential metabolic phenotyping.

Differential metabolic phenotyping based on substrate utilization was employed for the microbial differentiation of *Ochrobactrum* and typical and atypical *Brucella*. Bacterial growth was analyzed in a chemically defined minimal medium, which was adapted from those described by Gerhardt and Wilson (1) and Plommet (2).

For the preparation of 1 L of medium, add the substrates listed in the table below to 800 mL of distilled water. Adjust the pH with sodium hydroxide as necessary, and bring the volume up to 1 L. Sterilize the minimal medium by filtration through a 0.2 µm bottle-top-filter. Store the medium at 4°C in the dark for up to 4 weeks.

**Final concentration**	**Substance**	**Supplier**	**Article number**
5 g/L	NaCl	Carl Roth, Karlsruhe, Germany	9265.1
0.5 g/L	$(NH_4)_2SO_4$	Carl Roth, Karlsruhe, Germany	9212.3
0.01 g/L	$MgSO_4$	Carl Roth, Karlsruhe, Germany	P027.1
0.1 g/L	$MgCl_2$+6H_2O	Sigma Aldrich, Merck, Darmstadt, Germany	M2670
7 g/L	K_2HPO_4	Carl Roth, Karlsruhe, Germany	P749.1
3 g/L	KH_2PO_4	Carl Roth, Karlsruhe, Germany	3904.2
0.35 g/L	$NaHCO_3$	Carl Roth, Karlsruhe, Germany	0965.1
1 mg/L	$FeSO_4$	Alfa Aesar, Haverhill, MA, USA	33341
1 mg/L	$MnSO_4$	Alfa Aesar, Haverhill, MA, USA	14498
10 mL/L	BME Vitamins *	Merck, Darmstadt, Germany	B6891
0.4 µM	biotin	Merck, Darmstadt, Germany	B4501

* 100× composition: 0.1 g/L D-biotin, 0.1 g/L chlorine chloride, 0.1 g/L folic acid, 0.2 g/L myo-inositol, 0.1 g/L niacinamide, 0.1 g/L D-pantothenic acid·½Ca, 0.1 g/L pyridoxine HCl, 0.01 g/L riboflavin, 0.1 g/L thiamine HCl, 8.5 g/L sodium chloride

Prepare all growth substrates (listed in the table below) in minimal medium as 1 M stock solutions and adjust their pH with sodium hydroxide (dissolved in minimal medium) as necessary. Sterilize all substrate stocks by filtration using a 0.2 µm syringe filter and store them at 4°C in the dark for up to 4 weeks.

**Final concentration**	**Substance**	**Supplier**	**Article number**
20 mM	D-Glucose	Carl Roth, Karlsruhe, Germany	X997.1
20 mM	L-Rhamnose	Sigma Aldrich, Merck, Darmstadt, Germany	102578292
20 mM	D-Mannitol	Carl Roth, Karlsruhe, Germany	883.1
20 mM	D-Sorbitol	Carl Roth, Karlsruhe, Germany	6213.1
20 mM	Gluconic acid	Carl Roth, Karlsruhe, Germany	2621.1
20 mM	Adipic acid	Carl Roth, Karlsruhe, Germany	4475.2
20 mM	Citric acid	Carl Roth, Karlsruhe, Germany	4475.2
20 mM	Ectoine	Sigma Aldrich, Merck, Darmstadt, Germany	81619

The minimal medium was supplemented with a single substrate to a final concentration of 20 mM before inoculating with bacteria. Inoculum stocks were prepared in minimal medium.

References:

1. Gerhardt P, Wilson JB. The Nutrition of brucellae: Growth in Simple Chemically Defined Media. J Bacteriol. 1948; doi:10.1128/jb.56.1.17-24.1948.
2. Plommet M. Minimal requirements for growth of *Brucella suis* and other *Brucella* species. Zentralblatt für Bakteriologie. 1991; doi:10.1016/s0934-8840(11)80165-9.
