# Supplementary material for: A new member of the novel, non-core Brucella clade: An exotic frog isolate closely related to atypical Brucella isolates from recent human brucellosis cases in Australia
Source: BMC Microbiol. 2025 Dec 13;25:790. doi: 10.1186/s12866-025-04479-2 (PMC12701591; doi:10.1186/s12866-025-04479-2)
Supplement: Supplementary file 3 — Additional file 3. Minimum inhibitory concentrations (MICs) determined for Brucella sp. CVUAS_1139.3 using the E-test, compared with literature values. [file 12866_2025_4479_MOESM3_ESM.pdf]

Additional file 3 Minimum inhibitory concentrations (MICs) determined for *Brucella* sp. CVUAS_1139.3 using the E-test, compared with literature values.

	MICs (µg/mL)					Breakpoints	QC MICs (µg/mL)	
	Brucella sp. **CVUAS_1139.3**	Range of amphibian *Brucella* sp. [B]	Range for classical *Brucella* spp. [C]	*B. inopinata* BO1	*B. microti* * *B. microti*-like **	*Brucella* spp.	*Escherichia coli* ATCC 25922	
Antimicrobial agent	this study [A]	(1)	(2)	(3)	(4)	M45 (5)	this study [A]	M100 (6) M45 (5)
Ampicillin	≤ 8	2-8	n.a.	n.a.	n.a.	n.a.	6	2–8
Ampicillin-sulbactam	≤ 4[D]	n.a.	n.a.	n.a.	n.a.	n.a.	≤ 3[D]	2/1–8/4
Chloramphenicol	1	≤ 1-4	n.a.	n.a.	n.a.	n.a.	≤6	2-8
Ciprofloxacin	≤ 0.25	n.a.	n.a.	n.a.	n.a.	n.a.	≤ 0.008	0.004–0.016
**Doxycycline**	**≤ 0.75**	n.a.	0.06–0.5	0.12	S	S ≤ 1	3[E]	0.5–2
Erythromycin	≤ 3	2 to > 4	n.a.	n.a.	n.a.	n.a.	≤ 32	
**Gentamicin**	**≤ 1.5**	0.5–2	0.5–2	1	n.a.	S ≤ 4	0.5	0.25–1
Kanamycin	2	n.a.	n.a.	n.a.	n.a.	n.a.	≤ 2	1–4
Levofloxacin	0.19	n.a.	n.a.	n.a.	n.a.	n.a.	0.016	0.008–0.06
Norfloxacin	≤ 1	n.a.	n.a.	n.a.	n.a.	n.a.	≤ 0.047	0.03–0.12
Rifampin	8	n.a.	0.25–2	n.a.	S	n.a.	12	4–16
**Streptomycin**	**≤ 2**	n.a.	1–8	2	S	S ≤ 8	≤ 2	n.a.[F]
**Tetracycline**	**≤ 0.5**	0.25–2	0.06–0.5	0.25	n.a.	S ≤ 1	2	0.5–2
**Trimethoprim-sulfamethoxazole**	**1.5**[D]	≤ 0.25–2[D]	0.25–2[D]	≤ 0.5/9.5	S	S ≤ 2/38	0.19[D]	≤ 0.5/9.5

Reported MICs for other *Brucella* spp. and breakpoints from broth microdilution tests, as outlined by CLSI guidelines.

[A] highest MIC (µg/mL) documented within 48 h (20, 24, and 48 h) from two experiments in ambient air; [B] data of 27 amphibian isolates each tested in broth microdilution tests; [C] data of 35 isolates (*B. melitensis, B. suis*, and *B. abortus*) each tested in broth microdilution tests in ambient air at three test sites, while one test site reported data for 31 isolates only; [D] only the ampicillin and trimethoprim portion is shown; [E] values were repeatedly above the QC MIC, but the maximal MICs for *Brucella* sp. CVUAS_1139.3 remained below the breakpoint; [F] QC MICs for streptomycin reported by others were at 4-32 and 16 µg/mL (2, 7). * strain CCM4915; ** strain 17-2122-4144.

ATCC, American Type Culture Collection; CLSI, Clinical Laboratory Standards Institute; CVUAS, Chemisches und Veterinäruntersuchungsamt Stuttgart, Germany; MIC, minimum inhibitory concentration; S, susceptible; Trim-sulfa, Trimethoprim-sulfamethoxazole; QC, quality control; n.a., not available;

References:

1. Mühldorfer K, Wibbelt G, Szentiks CA, Fischer D, Scholz HC, Zschöck M, et al. The role of 'atypical' *Brucella* in amphibians: are we facing novel emerging pathogens? J Appl Microbiol. 2017; doi:10.1111/jam.13326.
2. Lonsway DR, Jevitt LA, Uhl JR, Cockerill FR, 3rd, Anderson ME, Sullivan MM, et al. Effect of carbon dioxide on broth microdilution susceptibility testing of *Brucella* spp. J Clin Microbiol. 2010; doi:10.1128/JCM.01860-09.
3. De BK, Stauffer L, Koylass MS, Sharp SE, Gee JE, Helsel LO, et al. Novel *Brucella* strain (BO1) associated with a prosthetic breast implant infection. J Clin Microbiol. 2008; doi:10.1128/JCM.01494-07.
4. Jay M, Girault G, Perrot L, Taunay B, Vuilmet T, Rossignol F, et al. Phenotypic and molecular characterization of *Brucella microti*-like bacteria from a domestic marsh frog (*Pelophylax ridibundus*). Frontiers in Veterinary Science. 2018; doi:10.3389/fvets.2018.00283.
5. CLSI. Methods for Antimicrobial Dilution and Disk Susceptibility Testing of Infrequently Isolated or Fastidious Bacteria. 3rd ed. Brewyn, PA: Clinical and Laboratory Standards Institute; 2016.
6. CLSI. Performance Standards for Antimicrobial Susceptibility Testing. 34th ed. Brewyn, PA: Clinical and Laboratory Standards Institute; 2024.
7. Brown SD, Traczewski MM. Broth microdilution susceptibility testing of *Brucella* species: quality control limits for ten antimicrobial agents against three standard quality control strains. J Clin Microbiol. 2005; doi:10.1128/JCM.43.11.5804-5807.2005.
