# Supplementary material for: A new member of the novel, non-core Brucella clade: An exotic frog isolate closely related to atypical Brucella isolates from recent human brucellosis cases in Australia
Source: BMC Microbiol. 2025 Dec 13;25:790. doi: 10.1186/s12866-025-04479-2 (PMC12701591; doi:10.1186/s12866-025-04479-2)
Supplement: Supplementary file 4 — Additional file 4. List of primers and probes used in this study. [file 12866_2025_4479_MOESM4_ESM.pdf]

Additional file 4 List of primers and probes used in this study.

PCR	Primer name	Nucleotide sequence (5′ → 3′)	Primer length	Amplicon (bp)	References
bcsp31	bcsp31-F	GCTCGGTTGCCAATATCAATGC	22	-	(1)
	bcsp31-R	GGGTAAAGCGTCGCCAGAAG	20		
	bcsp31-Probe	FAM-AAATCTTCCACCTTGCCCTTGCCATCA-BHQ	27		
IS711	IS711For	GCTTGAAGCTTGCGGACAGT	20		(2)
	IS711Rev	GGCCTACCGCTGCGAAT	17		
	IS711Probe	FAM-AAGCCAACACCCGGCCATTATGGT-BHQ1	24		
Bruce-ladder v2.0	BMEI0998f	ATCCTATTGCCCCGATAAGG	20	1,682	(3, 4)
	BMEI0997r	GCTTCGCATTTTCACTGTAGC	21		
	BMEI0535f	GCGCATTCTTCGGTTATGAA	20	450 / 1,320	
	BMEI0536r	CGCAGGCGAAAACAGCTATAA	21		
	BMEII0843f	TTTACACAGGCAATCCAGCA	20	1,071	
	BMEII0844r	GCGTCCAGTTGTTGTTGATG	20		
	BMEI1426f	TCGTCGGTGGACTGGATGAC	20	423 / 774	(4, 5)
	BMEI1427r	ATGGTCCGCAAGGTGCTTTT	20		
	BMEII0428f	GCCGCTATTATGTGGACTGG	20	587	(3, 4)
	BMEII0428r	AATGACTTCACGGTCGTTCG	20		
	BR0953f	GGAACACTACGCCACCTTGT	20	272	
	BR0953r	GATGGAGCAAACGCTGAAG	19		
	BMEI0752f	CAGGCAAACCCTCAGAAGC	19	218	
	BMEI0752r	GATGTGGTAACGCACACCAA	20		
	BMEII0987f	CGCAGACAGTGACCATCAAA	20	152	
	BMEII0987r	GTATTCAGCCCCCGTTACCT	20		
	Bmispec_f	AGATACTGGAACATAGCCCG	20	510	(4, 6)
	Bmispec_r	ATACTCAGGCAGGATACCGC	20		

References:

1. Probert WS, Schrader KN, Khuong NY, Bystrom SL, Graves MH. Real-time multiplex PCR assay for detection of *Brucella* spp., *B. abortus* and *B. melitensis*. J Clin Microbiol. 2004; doi:10.1128/JCM.42.3.1290-1293.2004.
2. Barkallah M, Gharbi Y, Hassena AB, Slima AB, Mallek Z, Gautier M, et al. Survey of infectious etiologies of bovine abortion during mid- to late gestation in dairy herds. PLoS One. 2014; doi:10.1371/journal.pone.0091549.
3. Garcia-Yoldi D, Marin CM, de Miguel MJ, Munoz PM, Vizmanos JL, Lopez-Goni I. Multiplex PCR assay for the identification and differentiation of all *Brucella* species and the vaccine strains *Brucella abortus* S19 and RB51 and *Brucella melitensis* Rev1. Clin Chem. 2006; doi:10.1373/clinchem.2005.062596.
4. Lopez-Goni I, Garcia-Yoldi D, Marin CM, de Miguel MJ, Barquero-Calvo E, Guzman-Verri C, et al. New Bruce-ladder multiplex PCR assay for the biovar typing of *Brucella suis* and the discrimination of *Brucella suis* and *Brucella canis*. Vet Microbiol. 2011; doi:10.1016/j.vetmic.2011.06.035.
5. Zygmunt MS, Blasco JM, Letesson JJ, Cloeckaert A, Moriyon I. DNA polymorphism analysis of *Brucella* lipopolysaccharide genes reveals marked differences in O-polysaccharide biosynthetic genes between smooth and rough *Brucella* species and novel species-specific markers. BMC Microbiol. 2009; doi:10.1186/1471-2180-9-92.
6. Scholz HC, Hubalek Z, Nesvadbova J, Tomaso H, Vergnaud G, Le Fleche P, et al. Isolation of *Brucella microti* from soil. Emerg Infect Dis. 2008; doi:10.3201/eid1408.080286.
