# Supplementary material for: A new member of the novel, non-core Brucella clade: An exotic frog isolate closely related to atypical Brucella isolates from recent human brucellosis cases in Australia
Source: BMC Microbiol. 2025 Dec 13;25:790. doi: 10.1186/s12866-025-04479-2 (PMC12701591; doi:10.1186/s12866-025-04479-2)
Supplement: Supplementary file 5 — Additional file 5. Sequence data used in in silico analyses. [file 12866_2025_4479_MOESM5_ESM.pdf]

Additional file 5 Sequence data used in *in silico* analyses.

***Brucella* spp.**	**Strain**	**Assemblies***
B. abortus bv. 1	544	GCF_000369945.1
B. abortus bv. 2	86/8/59	GCA_000740375.1
B. abortus bv. 3	Tulya	GCF_000157715.1
B. abortus bv. 4	292	GCF_000157695.1
B. abortus bv. 5	B3196	GCF_000163115.1
B. abortus bv. 6	870	GCF_000740215.1
B. abortus bv. 9	C68	GCF_000740195.1
B. canis	RM 6/66	GCA_000740335.1
B. ceti	B1/94	GCF_000158775.1
B. inopinata	FO700662	GCF_030270765.1
B. inopinata	BO1	GCF_000182725.1
B. melitensis bv. 1	16M	GCA_000007125.1
B. melitensis bv. 2	63/9	GCA_000022625.1
B. melitensis bv. 3	Ether	GCA_000740355.1
B. microti	CCM 4915	GCA_000022745.1
B. microti-like	17-2122-4144	GCF_902807045.1
B. neotomae	5K33	GCF_000158715.1
B. ovis	63/290	GCA_000016845.1
B. pinnipedialis	B2/94	GCA_000221005.1
B. suis bv. 1	1330	GCA_000007505.1
B. suis bv. 2	Thomsen	GCA_000018905.1
B. suis bv. 3	686	GCA_000740255.1
B. suis bv. 4	40	GCF_000160275.1
B. suis bv. 5	513	GCF_000157755.1
B. vulpis	F60	GCA_900000005.1
Brucella sp.	458	GCF_017797885.1
Brucella sp.	2280	GCF_009601725.1
Brucella sp.	6810	GCF_014495905.1
Brucella sp.	191011898	GCF_903131625.1
Brucella sp.	1410123041	GCF_900095155.1
Brucella sp.	09RB8910	GCF_001971805.1
Brucella sp.	09RB8913	GCF_009664925.1
Brucella sp.	09RB8918	GCF_009664935.1
Brucella sp.	B13-0095	GCF_001742815.1
Brucella sp.	BO2	GCF_015832115.1
Brucella sp.	BO3	GCF_014084005.1
***Brucella* sp.**	**CVUAS_1139.3**	**CP159274-CP159275***
Brucella sp.	NF2653	GCF_000177155.1
Brucella sp.	09RB8471	GCF_001971625.1
Brucella sp.	10RB9210	GCF_009664965.1
Brucella sp.	10RB9212	GCF_009665015.1
Brucella sp.	10RB9213	GCF_009664915.1
Brucella sp.	10RB9215	GCF_900092405.1

***Ochrobactrum* spp.**	**Strain**	**Assemblies***
O. anthropi	CIP 82.115	GCA_000017405.1
O. cytisi	IPA7.2	GCF_001876955.1
O. daejeonense	JCM 16234	GCA_012103095.1
O. gallinifaecis	ISO196	GCF_006476605.1
O. grignonense	OgA9a	GCF_002252505.1
O. haematophilum	DSM 22355	GCA_012103105.1
O. intermedium	CNS 2-75	GCA_900454225.1
O. lupini	LUP21	GCF_002252535.1
O. oryzae	NBRC 102588	GCA_012103035.1
O. pecoris	08RB2639	GCF_006376675.1
O. pituitosum	AA2	GCF_002025625.1
O. pseudintermedium	ASAG-D25	GCF_025118245.1
O. pseudogrignonense	K8	GCF_001652485.1
O. quorumnocens	A44	GCF_002278035.1
O. rhizosphaerae	PR17	GCF_002252475.1
O. soli	BO-7	GCF_003664555.1
O. thiophenivorans	DSM 7216	GCA_002252445.1
O. tritici	DSM 13340	GCA_012395245.1

***Brucella* spp.**	**Strain**	**Reads****
B. inopinata	FO700662	SRR22426907
Brucella sp.	458	SRR14074356
Brucella sp.	2280	SRR9620238
Brucella sp.	6810	SRR12630907
Brucella sp.	B13-0095	SRR3745442
Brucella sp.	BO2	SRR4038983
Brucella sp.	BO3	SRR10810417
***Brucella* sp.**	**CVUAS_1139.3**	**SRR28969979**

*Assemblies were used for the phylogenetic analysis based on average nucleotide identities and *in silico* bacterial characterization.
**Read data was used for the phylogenetic analysis based on single nucleotide polymorphisms.
***When this file was submitted, a GenBank/RefSeq Assembly ID had not yet been assigned.
bv., biovar
