# Supplementary material for: A new member of the novel, non-core Brucella clade: An exotic frog isolate closely related to atypical Brucella isolates from recent human brucellosis cases in Australia
Source: BMC Microbiol. 2025 Dec 13;25:790. doi: 10.1186/s12866-025-04479-2 (PMC12701591; doi:10.1186/s12866-025-04479-2)
Supplement: Supplementary file 6 — Additional file 6. Average nucleotide identities (ANIs) between Brucella sp. CVUAS_1139.3 and all analyzed Brucella spp. and Ochrobactrum spp. strains. [file 12866_2025_4479_MOESM6_ESM.pdf]

Additional file 6 Average nucleotide identities (ANIs) between *Brucella* sp. CVUAS_1139.3 and all analyzed *Brucella* spp. and *Ochrobactrum* spp. strains.

Brucella spp. strain	CVUAS_1139.3
**458**	**98.32**
09RB8913	98.14
09RB8918	98.12
2280	98.08
191011898	98.08
10RB9210	98.05
09RB8471	98.04
BO2	97.98
1410123041	97.95
FO700662	97.94
**BO1**	**97.90**
B13-0095	97.82
BO3	97.81
6810	97.79
17-2122-4144	97.62
CCM 4915	97.62
513	97.59
10RB9212	97.56
10RB9213	97.55
B2/94	97.55
10RB9215	97.54
5K33	97.54
40	97.53
686	97.52
1330	97.52
B1/94	97.52
C68	97.50
RM 6/66	97.50
63/9	97.49
870	97.49
09RB8910	97.49
Tulya	97.48
Thomsen	97.48
292	97.48
63/290	97.47
NF2653	97.47
86/8/59	97.47
544	97.45
Ether	97.45
**16M**	**97.43**
B3196	97.42
F60	97.17

Ochrobactrum spp. strain	CVUAS_1139.3
CNS 2-75	83.40
ASAG-D25	83.28
NBRC 102588	83.09
JCM 16234	82.80
DSM 13340	82.76
CIP 82.115	82.75
LUP21	82.75
08RB2639	82.62
IPA7.2	82.59
BO-7	82.36
DSM 22355	82.10
OgA9a	80.38
AA2	80.20
K8	80.20
PR17	79.75
A44	79.73
DSM 7216	79.47
ISO196	79.42

Pairwise ANIs were inferred with fastANI (v1.33) (1) and are listed in descending order. A complete overview of all ANI-values is provided in **Additional file 7,** a separate Excel file.

Reference:

1. Jain C, Rodriguez RL, Phillippy AM, Konstantinidis KT, Aluru S. High throughput ANI analysis of 90K prokaryotic genomes reveals clear species boundaries. Nature Communications. 2018; doi:10.1038/s41467-018-07641-9.
