# Supplementary material for: A new member of the novel, non-core Brucella clade: An exotic frog isolate closely related to atypical Brucella isolates from recent human brucellosis cases in Australia
Source: BMC Microbiol. 2025 Dec 13;25:790. doi: 10.1186/s12866-025-04479-2 (PMC12701591; doi:10.1186/s12866-025-04479-2)
Supplement: Supplementary file 8 — Additional file 8. MALDI Biotyper™ identification reports for Brucella sp. CVUAS_1139.3. [file 12866_2025_4479_MOESM8_ESM.pdf]

A

Projekt-ID: 240229-1835-1011014361 Projekt erzeugt am: 2024-02-29T18:36:27.897

Analyt- 1

Analytname: E11
Analytbeschreibung:
Analyt-ID: BfR-BR-00739 I
Analyt erzeugt am: 2024-02-29T18:36:27.928
Analyttyp: Standard
Identifikationsmethode: MALDI Biotyper MSP Identification Standard Method 1.1
Vorverarbeitungsmethode: MALDI Biotyper Preprocessing Standard Method 1.1
ACQ Methode: D:\Methods\flexControlMethods\MBT_FC.par
AutoXecute-Methode: MBT_AutoX
Konsistenzkategorie (basierend auf 2 besten Treffern): A
Verwendete MSP-Bibliothek(en): SR / contains 104 MSPs / 95ccc77e-d93e-44f0-8743-812aa90ff691 / 2017-09-29T14:53:19.245, BDAL / contains 11897 MSPs / ff6e1dd8-c047-4b48-bb64-1b3eae491247 / 2022-12-09T08:44:56.480, SR_BBFV / contains MSPs of the following four species/groups: Brucella melitensis, Burkholderia mallei/pseudomallei, Francisella tularensis, Vibrio cholerae. / cc6b3c5a-6cdb-43bc-a52b-6e76d11fe42f / 2017-09-29T14:53:19.248

Rang (Qualität)	Übereinstimmende Muster	Bewertungszahl	NCBI Code
1 (+++)	Brucella melitensis 6767	2.20	130738875
2 (+++)	Brucella melitensis 6273	2.20	130738875
3 (+++)	Brucella melitensis 5520	2.05	130738875
4 (+)	Brucella melitensis 6073	1.96	130738875
5 (+)	Brucella melitensis 6074	1.90	130738875
6 (-)	Brucella melitensis 5659	1.68	130738875
7 (-)	Ochrobactrum daejeonense DSM 26944T DSM	1.40	528
8 (-)	Streptococcus equi_ssp_zooepidemicus DSM 20727T DSM	1.34	40041

Ergebnistabelle für Analyt 1 – Fortsetzung auf nächster Seite

Report erzeugt am 2024-02-29T18:41:08 Nur für Forschungszwecke Seite 5 von 20

B

Projekt-ID: 240229-1835-1011014361 Projekt erzeugt am: 2024-02-29T18:36:27.897

Analyt- 2

Analytname: E12
Analytbeschreibung:
Analyt-ID: BfR-BR-00739 II
Analyt erzeugt am: 2024-02-29T18:36:27.928
Analyttyp: Standard
Identifikationsmethode: MALDI Biotyper MSP Identification Standard Method 1.1
Vorverarbeitungsmethode: MALDI Biotyper Preprocessing Standard Method 1.1
ACQ Methode: D:\Methods\flexControlMethods\MBT_FC.par
AutoXecute-Methode: MBT_AutoX
Konsistenzkategorie (basierend auf 2 besten Treffern): A
Verwendete MSP-Bibliothek(en): SR / contains 104 MSPs / 95ccc77e-d93e-44f0-8743-812aa90ff691 / 2017-09-29T14:53:19.245, BDAL / contains 11897 MSPs / ff6e1dd8-c047-4b48-bb64-1b3eae491247 / 2022-12-09T08:44:56.480, SR_BBFV / contains MSPs of the following four species/groups: Brucella melitensis, Burkholderia mallei/pseudomallei, Francisella tularensis, Vibrio cholerae. / cc6b3c5a-6cdb-43bc-a52b-6e76d11fe42f / 2017-09-29T14:53:19.248

Rang (Qualität)	Übereinstimmende Muster	Bewertungszahl	NCBI Code
1 (+++)	Brucella melitensis 6767	2.21	130738875
2 (+++)	Brucella melitensis 6273	2.13	130738875
3 (+++)	Brucella melitensis 5520	2.04	130738875
4 (+++)	Brucella melitensis 6074	2.01	130738875
5 (+)	Brucella melitensis 6073	1.96	130738875
6 (+)	Brucella melitensis 5659	1.70	130738875
7 (-)	Streptococcus equi_ssp_zooepidemicus ATCC 43079T THL	1.39	40041
8 (-)	Streptococcus equi_ssp_zooepidemicus DSM 20727T DSM	1.27	40041

Ergebnistabelle für Analyt 2 – Fortsetzung auf nächster Seite

Report erzeugt am 2024-02-29T18:41:08 Nur für Forschungszwecke Seite 7 von 20

Additional file 8 MALDI Biotyper™ identification reports for *Brucella* sp. CVUAS_1139.3. *Brucella* sp. CVUAS_1139.3 (BfR-BR-00739) was analyzed in duplicates using standard mass spectral libraries (BDAL and SD), identifying it as *Brucella melitensis* (**A**, **B**).
