# Supplementary material for: A new member of the novel, non-core Brucella clade: An exotic frog isolate closely related to atypical Brucella isolates from recent human brucellosis cases in Australia
Source: BMC Microbiol. 2025 Dec 13;25:790. doi: 10.1186/s12866-025-04479-2 (PMC12701591; doi:10.1186/s12866-025-04479-2)
Supplement: Supplementary file 9 — Additional file 9. Bruce‑ladder v2.0 results for Brucella sp. CVUAS_1139.3. [file 12866_2025_4479_MOESM9_ESM.pdf]

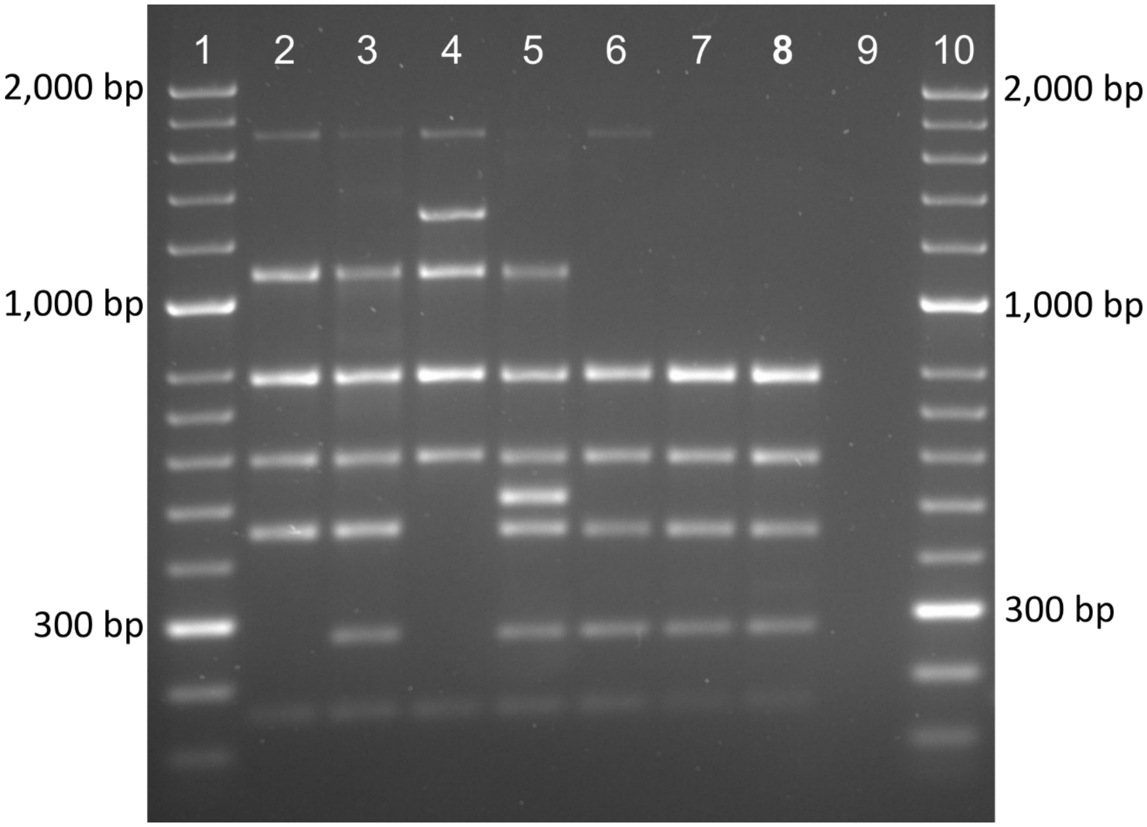

Additional file 9 Bruce-ladder v2.0 results for *Brucella* sp. CVUAS_1139.3. In the Bruce-ladder v2.0 assay, *Brucella* sp. CVUAS_1139.3 shows a band pattern identical to that of other exotic frog isolates. Lanes 1 and 10, 50 base pair (bp) HyperLadder™; lane 2, *B. melitensis*; lane 3, *B. suis*; lane 4, *B. ceti*; lane 5, *B. microti*; lane 6, *B. inopinata*; lane 7, *Brucella* sp. 09RB8471; lane 8, *Brucella* sp. CVUAS_1139.3; lane 9, non-template control.
