# Supplementary material for: A new member of the novel, non-core Brucella clade: An exotic frog isolate closely related to atypical Brucella isolates from recent human brucellosis cases in Australia
Source: BMC Microbiol. 2025 Dec 13;25:790. doi: 10.1186/s12866-025-04479-2 (PMC12701591; doi:10.1186/s12866-025-04479-2)
Supplement: Supplementary file 10 — Additional file 10. Differential metabolic phenotyping of exotic frog-derived Brucella sp. isolates. [file 12866_2025_4479_MOESM10_ESM.pdf]

	Brucella sp. 09RB8471	*Brucella* sp. 09RB8910	*Brucella* sp. 10RB9215	*Brucella* sp. 09RB8914	*Brucella* sp. 10RB9208	*Brucella* sp. 10RB9213	*Brucella* sp.10RB9214	*Brucella* sp. 09RB8913	***Brucella* sp. CVUAS_1139.3**
Minimal medium	0.00	0.00	0.00	0.00	0.01	0.00	0.00	0.01	0.00
Glucose	2.13	1.83	1.76	1.81	1.91	1.57	1.36	1.75	1.74
Rhamnose	0.00	0.00	0.00	0.00	0.00	1.59	0.00	0.01	1.84
Mannitol	0.00	0.00	0.01	0.01	0.00	0.01	0.00	0.01	0.02
Sorbitol	0.06	0.06	0.01	0.01	0.00	1.61	0.00	0.01	0.01
Gluconic acid	0.00	0.00	0.00	0.00	0.01	0.00	0.00	0.01	0.01
Adipic acid	1.46	1.53	1.37	1.31	1.56	1.37	0.85	1.40	1.38
Citric acid	0.00	0.00	0.00	0.00	0.00	0.00	0.00	0.02	0.02
Ectoine	0.69	1.19	1.73	0.57	0.54	1.11	0.69	0.48	0.02

African bullfrog isolates

bacterial growth: none | intermediate | good

maximal OD_{600}: 0.2, 0.5

Additional file 10 Differential metabolic phenotyping of exotic frog-derived *Brucella* sp. isolates. Inoculated single substrates (20 mM final concentration) were monitored over a period of 7 days. The data are presented as the mean maximal optical density at 600 nm (OD_{600}) of at least three independent experiments. Bacterial growth is categorized as good (black), intermediate (grey), or none (white) based on the following OD_{600} ranges: ≥ 0.5, 0.2–0.49 and < 0.19, respectively. For further details, refer to the Material and Methods section.
