# Supplementary material for: A new member of the novel, non-core Brucella clade: An exotic frog isolate closely related to atypical Brucella isolates from recent human brucellosis cases in Australia
Source: BMC Microbiol. 2025 Dec 13;25:790. doi: 10.1186/s12866-025-04479-2 (PMC12701591; doi:10.1186/s12866-025-04479-2)
Supplement: Supplementary file 11 — Additional file 11. Metadata for recently identified Australian Brucella sp. isolates that cluster within the novel, non-core clade. [file 12866_2025_4479_MOESM11_ESM.pdf]

Additional file 11 Metadata for recently identified Australian *Brucella* sp. isolates that cluster within the novel, non-core clade.

Metadata	*Brucella* sp. **458**	*Brucella* sp. **2280**	*Brucella* sp. **6810**
BioSample identifier *	SAMN18395631	SAMN12091575	SAMN15962648
Collection year	2021	2019	2020
Collected by	Queensland Health	Queensland Health	Queensland Health
Geographic location	Australia	Australia	Australia
Latitude and longitude	27 S 153 E	19 S 147 E	19.3 S 146.8 E
Host	*Homo sapiens*	*Homo sapiens*	*Homo sapiens*
Host disease	Granuloma	Brucellosis	Brucellosis
Isolation source / tissue	Lung tissue	Lymph node	Blood culture

*All information listed in this table was retrieved from the BioSample database at NCBI (accessed on March 2, 2024, at 4:30pm).
E, east; S, south
