# Supplementary material for: A new member of the novel, non-core Brucella clade: An exotic frog isolate closely related to atypical Brucella isolates from recent human brucellosis cases in Australia
Source: BMC Microbiol. 2025 Dec 13;25:790. doi: 10.1186/s12866-025-04479-2 (PMC12701591; doi:10.1186/s12866-025-04479-2)
Supplement: Supplementary file 12 — Additional file 12. Virulence factor genes and their coverages identified in a selection of novel Brucella strains. [file 12866_2025_4479_MOESM12_ESM.pdf]

Additional file 12 Virulence factor genes and their coverages identified in a selection of novel *Brucella* strains.

Gene \ *Brucella* spp.	CVUAS_1139.3	458	2280	6810	FO700662	BO1	BO2	BO3	B13-0095	09RB8471	09RB8910	09RB8913	10RB9210	10RB9213	191011898	1410123041
*acpXL**	100	100	100	100	100	100	100	100	100	100	100	100	100	100	100	100
BABS19	100	100	100	100	100	100	100	100	100	100	100	100	100	100	100	100
bmaA	50.38	50.38	-	-	50.6	-	50.19	-	-	-	67.55	-	-	50.75	-	-
bmaC	-	-	-	99.88	-	-	-	99.94	99.94	99.88	99.94	88.47	99.88	99.94	99.88	99.94
BPE005	100	100	100	100	100	100	100	100	100	100	100	100	100	100	100	100
BPE043	100	100	100	99.98	100	100	99.98	100	100	100	100	99.98	100	100	100	100
BPE123	100	100	100	100	100	100	100	100	100	100	100	100	100	100	100	100
BPE275	100	100	100	100	100	100	100	100	100	100	100	100	100	100	100	100
bspA	100	100	100	100	98.44	98.44	100	100	100	99.83	100	100	100	100	100	100
bspB	100	100	100	100	100	100	100	100	100	100	100	100	100	100	100	100
bspC	100	100	100	100	100	100	100	100	100	100	100	100	100	100	100	100
bspE	100	100	100	100	100	100	100	100	100	100	100	100	100	100	100	100
bspF	100	100	100	100	100	100	100	100	100	100	100	100	100	100	100	100
bspJ	100	100	100	100	100	100	100	100	100	100	100	100	100	100	100	100
bspL	100	100	100	100	100	100	100	100	100	100	100	100	100	100	100	100
btaE	-	-	-	71.91	-	-	-	80.36	55.59	63.44	54.95	63.44	63.44	63.42	63.44	54.95
btaF	-	-	-	73.78	-	-	-	70.04	-	72.36	72.36	68.09	73.78	73.78	73.69	-
btpA	100	-	-	-	-	-	-	-	-	-	-	-	-	-	-	-
bvrR	100	100	100	100	100	100	100	100	100	100	100	100	100	100	100	100
bvrS	100	100	100	100	100	100	100	100	100	100	100	100	100	100	100	100
cgs	100	100	100	100	100	100	100	100	100	100	100	100	100	100	100	100
dhbA	100	100	100	100	100	100	100	100	100	100	100	100	100	100	100	100
dhbB	100	100	99.31	99.31	100	100	100	100	100	100	100	100	100	100	100	100
dhbC	100	100	100	100	100	100	100	100	100	100	100	100	100	100	100	100
dhbE	100	100	100	100	100	100	100	100	100	100	100	100	100	100	100	99.94
entD	100	100	100	100	100	100	100	100	100	100	100	100	100	100	100	100
fabZ	100	100	100	100	100	100	100	100	-	100	100	100	100	100	100	100
gmd	-	-	-	-	100	100	-	-	-	-	-	-	-	-	-	-
htrB	100	100	100	100	100	100	100	100	100	100	100	100	100	100	100	100
kdsA	99.76	99.76	100	100	99.76	99.76	99.76	100	-	99.76	100	99.76	99.76	99.76	99.76	100
kdsB	100	100	100	100	100	100	100	100	100	100	100	100	100	100	100	100
lpsA	100	99.95	99.95	99.95	100	100	100	99.95	99.95	100	99.95	99.95	100	100	99.95	100
lpsB	100	100	100	100	100	100	100	100	100	100	100	100	100	100	100	100
lpxA	100	100	100	100	100	100	100	100	-	100	100	100	100	100	100	100
lpxB	100	100	100	100	100	100	100	100	-	100	100	100	100	100	100	100
lpxC	100	100	100	100	100	100	100	100	100	100	100	100	100	100	100	100
lpxD	100	100	100	100	100	100	100	100	-	99.91	100	100	100	100	100	100
lpxE	100	100	100	100	100	100	100	100	100	100	100	100	100	100	100	100
lpxK	100	100	100	100	100	100	100	100	100	100	100	100	100	100	100	100
manA$_{O\text{-}Ag}$	-	-	100	100	100	100	-	-	-	-	-	-	-	-	-	-
manB$_{core}$	100	100	100	100	100	100	100	100	100	100	100	100	100	100	100	100
manC$_{core}$	100	100	100	100	100	100	100	100	100	100	100	100	100	100	100	100
manC$_{O\text{-}Ag}$	-	-	100	100	100	100	-	-	-	-	-	-	-	-	-	-
per	-	-	-	-	100	100	-	-	-	-	-	-	-	-	-	-

Gene \ *Brucella* spp.	CVUAS_1139.3	458	2280	6810	FO700662	BO1	BO2	BO3	B13-0095	09RB8471	09RB8910	09RB8913	10RB9210	10RB9213	191011898	1410123041
pgm	100	100	100	100	100	100	100	100	100	100	100	100	100	100	100	100
pmm	-	-	86.97	100	100	100	-	-	-	-	-	-	-	-	-	-
ricA	100	100	100	100	100	100	100	100	100	100	100	100	100	99.62	100	100
ugpB	100	100	100	100	100	100	100	100	100	100	100	100	100	100	100	100
vceA	100	99.69	99.69	100	99.69	99.69	99.69	100	100	-	100	100	-	100	100	100
vceC	98.81	98.81	100	100	100	99.92	98.81	100	100	98.73	100	98.81	98.81	99.05	98.81	98.81
vibH	100	100	100	100	100	100	100	100	100	100	100	100	100	100	100	100
virB1	100	100	100	100	100	100	100	100	100	100	100	100	100	100	100	100
virB10	98.95	98.95	98.95	98.95	98.95	98.95	98.95	98.95	98.95	98.95	99.74	98.95	98.95	100	98.95	98.95
virB11	100	100	100	100	100	100	100	100	100	100	100	100	100	100	100	100
virB12	100	99.81	100	99.81	99.81	99.81	100	99.81	99.81	99.81	100	99.81	99.81	100	99.81	99.81
virB2	96.86	96.86	96.86	96.86	96.86	96.86	96.86	96.86	96.86	96.86	98.11	96.86	96.86	100	96.86	96.86
virB3	100	100	100	100	100	100	100	100	100	100	100	100	100	100	100	100
virB4	100	100	100	100	100	100	100	100	100	100	100	100	100	100	100	100
virB5	100	100	100	100	100	100	100	100	100	100	99.58	100	100	99.3	100	100
virB6	100	100	100	100	100	100	100	100	100	100	100	100	100	100	100	100
virB7	100	100	100	100	100	100	100	100	100	100	100	100	100	100	100	100
virB8	100	100	100	100	100	100	100	100	100	100	100	100	100	100	100	100
virB9	100	100	100	100	100	100	100	100	100	100	100	100	100	100	100	100
waaA	100	100	100	100	100	100	100	100	100	100	100	100	100	100	100	100
wbdA	-	-	-	-	100	100	-	-	-	-	-	-	-	-	-	-
wbkA	-	-	-	-	100	99.2	-	-	-	-	-	-	-	-	-	-
wbkB	-	-	-	-	100	100	-	-	-	-	-	-	-	-	-	-
wbkC	-	-	-	-	100	100	-	-	-	-	-	-	-	-	-	-
wboA	-	-	-	-	100	100	-	-	-	-	-	-	-	-	-	-
wbpL	100	100	100	100	100	100	100	100	-	100	100	100	100	100	100	100
wbpZ	-	-	100	100	100	100	-	-	-	-	-	-	-	-	-	-
wzm	-	-	-	-	100	100	-	-	-	-	-	-	-	-	-	-
wzt	-	-	-	-	100	100	-	-	-	-	-	-	-	-	-	-

This table spans two pages, so the header is repeated to improve readability. List of all virulence genes identified in the analyzed strains and their relative coverage (%) of the respective reference gene as determined with the BakCharak pipeline (v3.1.2) (1) using ABRicate (v1.0.1) (2) with the virulence factor database vfdb_brucella_setB (v.2022-08-26) and default parameters (minimum coverage of 50%) (3). *The *acpXL* gene was found twice per strain, each with 100% coverage of the reference gene sequence. **Bold gene name**, gene is involved in lipopolysaccharide (LPS) synthesis. Underlined number, the gene was found twice with different coverages. -, no value reported. A complete overview is provided in **Additional file 13** in a separate Excel file.

References:

1. Deneke C. Bakcharak: Bakterial characterization of food-borne pathogens. Github https://gitlab.com/bfr_bioinformatics/bakcharak.
2. Seemann T. ABRicate. Github https://github.com/tseemann/abricate.
3. Chen L, Yang J, Yu J, Yao Z, Sun L, Shen Y, et al. VFDB: a reference database for bacterial virulence factors. Nucleic Acids Res. 2005; doi:10.1093/nar/gki008.
