# Supplementary material for: A new member of the novel, non-core Brucella clade: An exotic frog isolate closely related to atypical Brucella isolates from recent human brucellosis cases in Australia
Source: BMC Microbiol. 2025 Dec 13;25:790. doi: 10.1186/s12866-025-04479-2 (PMC12701591; doi:10.1186/s12866-025-04479-2)
Supplement: Supplementary file 14 — Additional file 14. Intracellular replication of novel and classical Brucella spp. in human THP-1 macrophage-like cells. [file 12866_2025_4479_MOESM14_ESM.pdf]

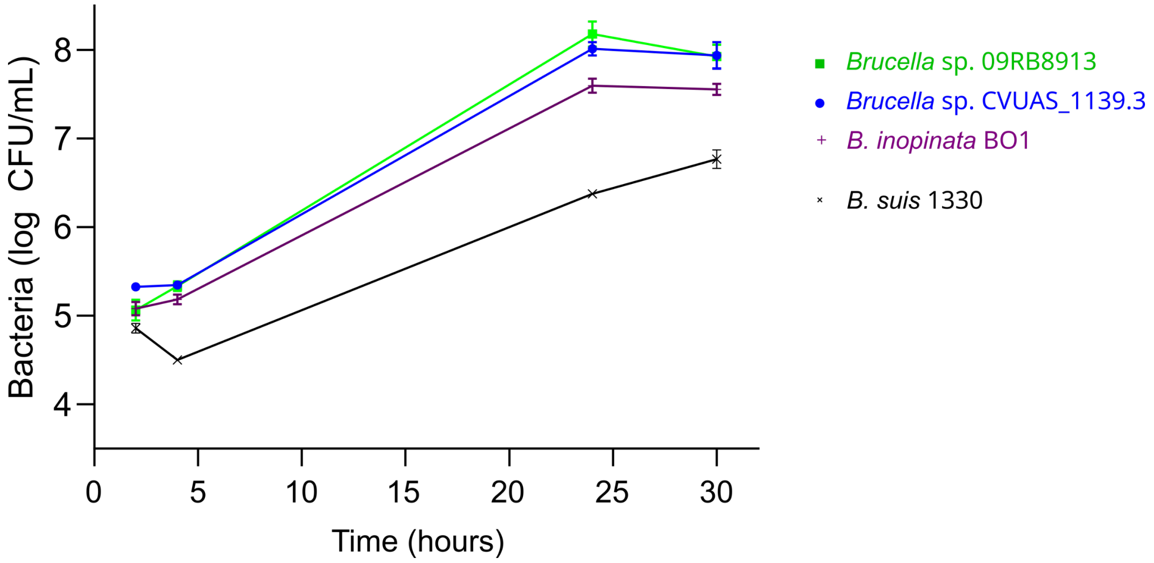

Additional file 14 Intracellular replication of novel and classical *Brucella* spp. in human THP-1 macrophage-like cells. Intracellular replication of *Brucella* sp. CVUAS_1139.3 was compared with novel *Brucella* spp., including the African bullfrog strain *Brucella* sp. 09RB8913 and *B. inopinata* BO1, as well as the classical *Brucella* representative *B. suis* 1330 in human THP-1 macrophage-like cells. The experiment was performed twice, with technical triplicates in each case, resulting in comparable results. The results from the second experiment are shown, presented as the mean ± standard deviation. Error bars smaller than the symbol are not shown. CFU, colony-forming unit.
